# Ag‐Co_3_O_4_‐CoOOH‐Nanowires Tandem Catalyst for Efficient Electrocatalytic Conversion of Nitrate to Ammonia at Low Overpotential via Triple Reactions

**DOI:** 10.1002/advs.202303789

**Published:** 2023-10-11

**Authors:** Shilu Wu, Yingyang Jiang, Wenjie Luo, Peng Xu, Longlong Huang, Yiwen Du, Hui Wang, Xuemei Zhou, Yongjie Ge, Jinjie Qian, Huagui Nie, Zhi Yang

**Affiliations:** ^1^ Key Laboratory of Carbon Materials of Zhejiang College of Chemistry and Materials Engineering Wenzhou University Wenzhou 325035 P. R. China

**Keywords:** Ag/Co_3_O_4_/CoOOH NWs, ammonia, electrocatalysis, nitrate reduction, tandem catalysis, triple reactions

## Abstract

The electrocatalytic conversion of nitrate (NO_3_‾) to NH_3_ (NO_3_RR) offers a promising alternative to the Haber–Bosch process. However, the overall kinetic rate of NO_3_RR is plagued by the complex proton‐assisted multiple‐electron transfer process. Herein, Ag/Co_3_O_4_/CoOOH nanowires (i‐Ag/Co_3_O_4_ NWs) tandem catalyst is designed to optimize the kinetic rate of intermediate reaction for NO_3_RR simultaneously. The authors proved that NO_3_‾ ions are reduced to NO_2_‾ preferentially on Ag phases and then NO_2_‾ to NO on Co_3_O_4_ phases. The CoOOH phases catalyze NO reduction to NH_3_ via NH_2_OH intermediate. This unique catalyst efficiently converts NO_3_‾ to NH_3_ through a triple reaction with a high Faradaic efficiency (FE) of 94.3% and a high NH_3_ yield rate of 253.7 μmol h^−1^ cm^−2^ in 1 M KOH and 0.1 M KNO_3_ solution at ‒0.25 V versus RHE. The kinetic studies demonstrate that converting NH_2_OH into NH_3_ is the rate‐determining step (RDS) with an energy barrier of 0.151 eV over i‐Ag/Co_3_O_4_ NWs. Further applying i‐Ag/Co_3_O_4 _NWs as the cathode material, a novel Zn‐nitrate battery exhibits a power density of 2.56 mW cm^−2^ and an FE of 91.4% for NH_3 _production.

## Graphical abstract

The Ag/Co_3_O_4_/CoOOH nanowires (i‐Ag/Co_3_O_4_ NWs) tandem catalyst is designed to optimize the kinetic rate of intermediate reaction for NO_3_RR simultaneously. The kinetic studies reveal that NO_3_‾ ions are reduced to NO_2_‾ preferentially on Ag phases, and then the NO_2_‾ intermediates are converted to NO on Co_3_O_4_ phases. The CoOOH phases catalyze NO reduction to NH_3_ via the NH_2_OH intermediate.

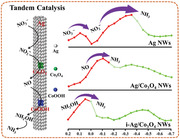

## 1 Introduction

Ammonia (NH_3_) is an indispensable chemical for fertilizer, textiles, pharmaceuticals, etc. It is also deemed a clean energy carrier owing to being hydrogen‐rich but carbon‐free.^[^ ^1^, ^2^, ^3^ ^]^ Currently, the industrial‐scale NH_3_ synthesis relies on the energy‐intensive Haber–Bosch process, which is the reaction between dinitrogen (N_2_) and hydrogen (H_2_) under high temperature (400–500 °C) and high pressure (150–300 atm).^[^ ^4^, ^5^ ^]^ From an energy‐saving viewpoint, electrocatalytic reduction of N_2_ to NH_3_ (NRR) under ambient conditions has been extensively explored over the past few years to replace the Haber–Bosch process.^[^ ^6^, ^7^ ^]^ However, NRR suffers low selectivity and activity due to the highly stable N≡N triple bond (941 kJ mol^−1^) and low water solubility.^[^ ^8^, ^9^ ^]^ To cover the shortage, electrocatalytic nitrate (NO_3_‾) reduction to ammonia (NO_3_RR) is desirable because the NO_3_‾ exhibits comparatively low dissociation energy of the N═O bond (204 kJ mol^−1^).^[^ ^10^, ^11^ ^]^ Also, NO_3_‾ is widely abundant as pollution in agricultural and industrial wastewater.^[^ ^12^ ^]^ Therefore, developing NO_3_RR opens a green route to synthesize NH_3_ and can address environmental pollution problems.

The NO_3_RR is a complex 8e^‒^ transfer process, the conversion process of NO_3_‾ to NH_3_ will undergo many intermedia reactions such as NO_3_‾ → *NO_3_‾ → *NO_2_‾ → *NO → **^……^** → *NH_3_ → NH_3_ (* denotes a surface‐adsorbed species), which remarkably lowers the overall kinetic rate.^[^ ^13^, ^14^ ^]^ In this regard, the rational design and development of efficient catalysts with high activity and efficiency toward the NO_3_RR are highly desirable. So far, a series of electrocatalysts based on noble metals,^[^ ^15^, ^16^ ^]^ transition metals,^[^ ^17^ ^]^ bimetallic materials,^[^ ^18^, ^19^, ^20^ ^]^ and metal oxide^[^ ^21^, ^22^, ^23^ ^]^ have been developed to convert NO_3_‾ into NH_3_. Although these strategies have improved the conversion efficiency of NO_3_‾ to NH_3_, the overall kinetic rate of NO_3_RR is still plagued by the complex reaction path because it is difficult to accelerate the kinetic rate of each step in the NO_3_RR process by a monofunctional catalyst. “Tandem catalysis” has been successfully reported for complex multi‐electron transfer reactions, such as the CO_2_ reduction reaction, a strategy based on the synergistic action of multiple catalyst components that can efficiently catalyze each step.^[^ ^11^, ^24^, ^25^, ^26^, ^27^ ^]^ More recently, researchers have designed the Cu/Co‐based phase tandem catalyst to reduce NO_3_‾, which can be combined to “working‐in‐tandem” for rapid NH_3_ synthesis.^[^ ^23^, ^28^, ^29^ ^]^ In these studies, the NO_3_RR was separated into two stages to alleviate the kinetic barrier. The first stage involved the catalysis of the NO_3_‾ → NO_2_‾ reaction by one type of catalyst, while the second stage involved the catalysis of the NO_2_‾ → NH_3_ reaction (NO_2_RR) by a different kind of catalyst. Regrettably, the NO_2_RR is also a complicated multi‐electron transfer process that involves six‐electron transfer steps and requires deoxidation and hydrogenation reactions.^[^ ^40^ ^]^ Thus, developing multiple tandem catalysts is necessary for efficient NO_3_RR, but it has not gotten enough attention.

Because the reactivity of NO_2_‾ is higher than that of the stable NO_3_‾, it is generally more accessible to the reduction of NO_2_‾ on most metal surfaces. Therefore, it is vital to the NO_3_RR process to choose a suitable catalyst for enhancing the conversion of NO_3_‾ into NO_2_‾. Among the metal catalysts, silver (Ag)‐based catalysts exhibit the most vigorous electrocatalytic activity for the conversion of NO_3_‾ into NO_2_‾.^[^ ^30^ ^]^ In addition, given that 1D nanowire structures have attractive superiorities in electrocatalysis for their outstanding conductivity and effective avoidance of aggregation, dissolution, and Ostwald ripening of catalysts.^[^ ^31^, ^32^, ^33^, ^34^ ^]^ Thus, we chose the silver nanowires (Ag NWs) as a template to synthesize the tandem catalyst. The employment of Co_3_O_4_ as a sub‐component of the tandem catalysts is due to the high selectivity of Co‐based catalysts for NH_3_ synthesis, especially for the conversion of NO_2_‾ to NH_3_.^[^ ^35^, ^36^, ^37^ ^]^ However, the catalytic performance of Co_3_O_4_ is still limited by the low electrical conductivity due to the inherently large bandgap.^[^ ^38^, ^39^ ^]^ On the other hand, it is challenging for a single Co_3_O_4_ phase to enhance all the intermediate steps of the NO_2_RR process simultaneously. Actually, it is interesting to note that CoOOH has better conductivity relative to Co_3_O_4_ and is a promising candidate for hydrogen evolution reactions (HER).^[^ ^40^ ^]^ The previous reports have demonstrated that catalysts that promote the HER are typically advantageous for the hydrogenation steps in the NO_3_RR process.^[^ ^41^, ^42^, ^43^, ^44^ ^]^ This can lead to an accelerated overall kinetic rate of the NO_3_RR process.

Triggered by the above discussion, we developed Ag/Co_3_O_4_/CoOOH NWs (i‐Ag/Co_3_O_4_ NWs) as the tandem catalyst for achieving an efficient electrochemical reduction of NO_3_‾ to NH_3_ via triple reactions. Our electrocatalytic tests, kinetic studies, and in situ infrared spectra reveal that at low overpotentials, the Ag phases catalyzed the NO_3_‾ convert into NO_2_‾, while the Co_3_O_4_ phases preferentially catalyzed NO_2_‾ reduction to NO, the subsequent reaction of NO hydrogenation to NH_3_ is mainly catalyzed by CoOOH. In situ Raman studies indicate that the catalytic effect of CoOOH on the NO → NH_3_ process is attributed to its dehydrogenation reaction in the NO_3_RR process, which provided sufficient protons for the hydrogenation of NO. As a result, the i‐Ag/Co_3_O_4_ NWs tandem catalyst could convert NO_3_‾ to NH_3_ with a high faraday efficiency (FE) of 94.3%, a high NH_3_ yield rate of 253.7 µmol h^−1^ cm^−2^ at ‒0.25 V versus RHE (Reversible hydrogen electrode) in 1 m KOH and 0.1 m KNO_3_ solution. Considering the outstanding catalytic NO_3_RR activity and selectivity of i‐Ag/Co_3_O_4_ NWs, a novel Zn‐nitrate battery with i‐Ag/Co_3_O_4_ NWs as the cathode and Zn plate as the anode with an open circuit potential of 1.32 V was developed. This Zn‐nitrate battery also exhibits a power density of 2.56 mW cm^−2^ and high FE of 91.4% for NH_3_ production with good electrochemical stability.

## 2 Results and Discussion

**Figure 1:**
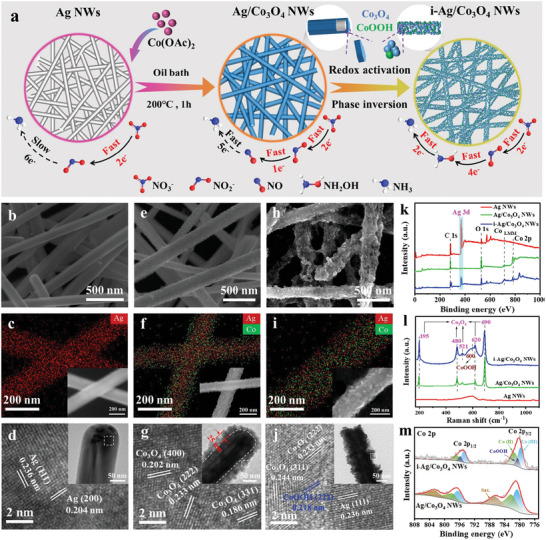
Structural characterizations of catalysts. a) Schematic illustration of the synthetic process of the i‐Ag/Co_3_O_4_ NWs tandem catalyst. SEM images of b) Ag NWs, e) Ag/Co_3_O_4_ NWs, and h) i‐Ag/Co_3_O_4_ NWs. EDX elemental mapping images of the c) Ag NWs, f) Ag/Co_3_O_4_ NWs, and i) i‐Ag/Co_3_O_4_ NWs. Typical TEM images of d) Ag NWs, g) Ag/Co_3_O_4_ NWs, and j) i‐Ag/Co_3_O_4_ NWs. k) XPS spectra, l) The Raman spectra, m) XPS spectra of Co 2p of the as‐synthesized catalysts.

The synthesis of the Ag/Co_3_O_4_/CoOOH NWs tandem catalyst is schematically illustrated in **Figure** **1**a. First, the Ag NWs were prepared by the polyol reduction method.^[^ ^45^, ^46^ ^]^ Figure 1b and Figure S1 (Supporting Information) demonstrate that the as‐prepared Ag NWs with an average diameter of 157.48 nm and an average length of 22.08 µm have a smooth and clean surface without any coating layer. Energy dispersive X‐ray spectrometers (EDX) elemental mapping images reveal the uniform distribution of Ag in the Ag NWs (Figure 1c). The XRD pattern of Ag NWs exhibits the characteristic peaks of Ag attributed to (111), (200), (220), and (311) facets (Figure S2, Supporting Information). The high‐resolution transmission electron microscopy (HRTEM) image of Ag NWs presents the lattice fringe distance of 0.236 and 0.204 nm (Figure 1d), which belongs to the (111) and (200) planes of Ag NWs.

Subsequently, the as‐synthesized Ag NWs were dispersed 10 mL of 30 mm Co(OAc)_2_·4H_2_O oleylamine solution and heated at 200 °C for 1 h under vigorous stirring in the Ar atmosphere, and the Ag/Co‐based phase NWs were obtained. Obviously, the smooth and clean surface of Ag NWs converted into a rough surface and evenly adhered with many nanoparticles (Figure 1e). This result preliminarily indicates that the Co‐based phase is successfully attached to the Ag NWs surface. In addition, Ag/Co‐based phase NWs have an average diameter of 191.64 nm and an average length of 21.49 µm (Figure S3, Supporting Information), indicating that the preparation process does not affect the nanowire length. The XRD pattern not only displays the characteristics of Ag but also exhibits the characteristic peaks of Co_3_O_4_ attributed to (111), (311), (222), (400), (511) and (440) facets (Figure S2, Supporting Information). This result demonstrates the Co‐based phase shell is the Co_3_O_4_ phase and is further verified by the Raman spectra. As shown in Figure 1l, the Raman spectrum of Ag/Co_3_O_4_ NWs shows five more peaks at 690, 620, 521, 480, and 195 cm^−1^ than that of Ag NWs, corresponding to the typical Raman‐active modes of A_1g_, F_2g_, F_2g_, E_g_, and F_2g_ of Co_3_O_4_, respectively.^[^ ^23^, ^38^ ^]^ EDX elemental mapping image verifies Ag core and Co_3_O_4_ shell distribution in the Ag/Co_3_O_4_ NWs (Figure 1f). This result was further confirmed by the TEM image illustrated in the inset of Figure 1g. The Ag/Co_3_O_4_ NWs show a core‐shell structure with a 113 nm core and 25 nm shell. The lattice fringes with a distance of 0.202, 0.233, and 0.186 nm are observed in the HRTEM image of the Ag/Co_3_O_4_ NWs shell, which corresponds to the (400), (222), and (331) plane of Co_3_O_4_ (Figure 1g), respectively.

Finally, we utilize the electrochemical activation strategy to treat the core‐shell Ag/Co_3_O_4_ NWs (labeled as i‐Ag/Co_3_O_4_ NWs). In brief, the Ag/Co_3_O_4_ NWs were polarized by cyclic voltammetry (CV) range from 0.05 to 2.05 V versus RHE in an Ar‐saturated 1 m KOH solution for 4 cycles (Figure S4, Supporting Information). The i‐Ag/Co_3_O_4_ NWs have a rougher and looser surface compared to Ag/Co_3_O_4_ NWs (Figure 1h). The SEM comparison demonstrated that the electrochemical activation treatment could effectively engrave the Co_3_O_4_ phase to increase the surface area, resulting in enhanced electrochemical performance.^[^ ^47^ ^]^ In addition, the electrochemical activation process also does not affect the length of the nanowires (Figure S5, Supporting Information). The EDX elemental mapping image showed the uniform distribution of Ag and Co in the i‐Ag/Co_3_O_4_ NWs, indicating that the Ag in the core migrates to the outer shell after electrochemical activation treatment (Figure 1i). X‐ray photoelectron spectroscopy (XPS) studies can further confirm this migration process. As shown in Figure 1k, the XPS spectrum of Ag NWs shows a strong peak at 367.8 and 373.8 eV belonging to Ag 3d. However, the XPS spectrum of Ag/Co_3_O_4_ NWs exhibits a fragile Ag 3d signal because the Ag core is predominantly enveloped by the Co_3_O_4_ shell. In contrast, after electrochemical activation treatment, the XPS signal for Ag 3d was re‐enhanced in the XPS spectrum of i‐Ag/Co_3_O_4_ NWs. Unlike Ag/Co_3_O_4_ NWs, the HRTEM image at the edge of i‐Ag/Co_3_O_4_ NWs shows a typical (111) facet of Ag (Figure 1j). These results demonstrate that the electrochemical activation process migrates Ag atoms outward to the nanowire surface and achieves an atomic‐scale Ag/Co interface, which will be more conducive to the conversion of NO_3_‾ to NO_2_‾ step of NO_3_RR as discussed below.

After electrochemical activation treatment, distinct peaks emerged in the XRD pattern of i‐Ag/Co_3_O_4_ NWs at 2θ = 20.3°, corresponding to (111) facets of CoOOH (Figure S2, Supporting Information). This discovery implies the emergence of a novel CoOOH phase, which is additionally corroborated by the findings from HRTEM analysis and Raman spectra. As shown in Figure 1j, in addition to the identification of the (311) and (222) crystal planes associated with Co_3_O_4_ and the (111) facet of Ag, the (222) crystal plane attributed to CoOOH was observed. As shown in Figure 1l, the Raman spectrum of i‐Ag/Co_3_O_4_ NWs exhibits a new peak at 600 cm^−1^, attributing to the Raman‐active mode of A_1g_ of CoOOH.^[^ ^48^, ^49^ ^]^ As observed in Figure 1m, the XPS spectrum for high‐resolution Co 2p in Ag/Co_3_O_4_ NWs and i‐Ag/Co_3_O_4_ NWs are segmented into four prominent peaks at 797.5, 795.9, 782.4, and 780.8 eV, which belonged to Co^2+^ 2p_1/2_, Co^3+^ 2p_1/2_, Co^2+^ 2p_3/2_, and Co^3+^ 2p_3/2_, respectively.^[^ ^47^ ^]^ Binding energies at 786.7 and 805.3 eV are assigned to satellite peaks.^[^ ^50^ ^]^ After electrochemical activation treatment, the XPS signals attributed to the CoOOH are observed in the XPS spectrum of i‐Ag/Co_3_O_4_ NWs. These observations prove that the Ag/Co_3_O_4_/CoOOH tandem catalyst with large surface areas and atomic‐scale Ag/Co interfaces was successfully synthesized by electrochemical activation of core‐shell Ag/Co_3_O_4_ NWs.

**Figure 2:**
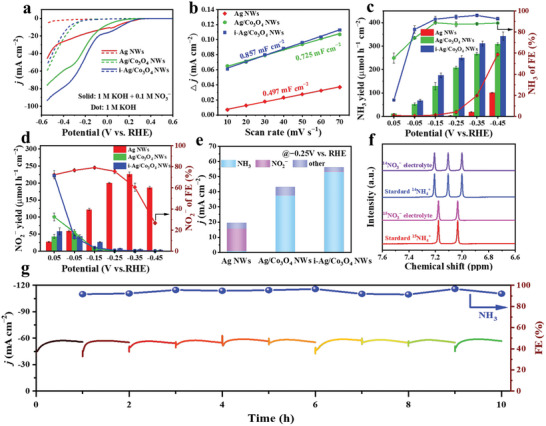
NO_3_^−^‐to‐NH_3_ conversion performance. a) LSV curves of the as‐synthesized catalysts in 1 m KOH electrolyte with and without KNO_3_. b) The determination of double‐layer capacitance for each catalyst. c) NH_3_ yields and FEs of Ag NWs, Ag/Co_3_O_4_ NWs, and i‐Ag/Co_3_O_4_ NWs. d) NO_2_^−^ yields and FEs of Ag NWs, Ag/Co_3_O_4_ NWs, and i‐Ag/Co_3_O_4_ NWs. e) The current densities of NH_3_, NO_2_^−^, and other products were collected at −0.25 V versus RHE. f) ^1^H NMR spectra of ^15^NH_4_Cl and ^14^NH_4_Cl standard solutions and electrolyte after the NO_3_RR electrolysis using K^15^NO_3_ and K^14^NO_3_ as the nitrogen source. g) Chronoamperometric stability test at −0.25 V versus RHE and corresponding NH_3_ FEs of i‐Ag/Co_3_O_4_ NWs.

The electrocatalytic NO_3_RR activity of as‐synthesized catalysts was evaluated in 1 m NaOH and 0.1 m KNO_3_ solution. The linear sweep voltammetry (LSV) curve of i‐Ag/Co_3_O_4_ NWs exhibits the highest current density in the entire test potential range (**Figure** **2**a), indicating the catalytic activity of i‐Ag/Co_3_O_4_ NWs for NO_3_RR outperformed that of Ag NWs and Ag/Co_3_O_4_ NWs. All catalysts exhibit a damped current density without NO_3_‾, implying a low contribution of the HER to the total current density in the nitrate solution.^[^ ^51^ ^]^ In addition, the Tafel slope of the HER shows that the catalytic activities of Co‐based electrocatalysts outclass that of Ag NWs (Figure S6, Supporting Information), and the improved HER performance may benefit the hydrogenation step of NO_3_RR.^[^ ^52^ ^]^ The double‐layer capacitances (C_dl_) were measured to assess the electrochemical surface areas (ECSA) of as‐synthesized catalysts (Figure S7, Supporting Information). As shown in Figure 2b, the C_dl_ of Ag NWs, Ag/Co_3_O_4_ NWs, and i‐Ag/Co_3_O_4_ NWs are 0.497, 0.725, and 0.857 mF cm^−2^, respectively, manifesting that i‐Ag/Co_3_O_4_ NWs possess highest ECSA. A larger ECSA can expose more active sites, which can endow the i‐Ag/Co_3_O_4_ NWs with high NO_3_RR catalytic activity, in agreement with the SEM results.

The yield rate and Faradaic efficiencies(FEs) for the products (NH_3_ and NO_2_‾) of as‐synthesized catalysts were probed in the 1 m NaOH and 0.1 m KNO_3_ electrolyte at different applied potentials (Figure 2c,d). The generated products were quantified by ultraviolet‐visible (UV–vis) spectrophotometry (Figure S8, Supporting Information). The time‐dependent current density (*j‐t*) curves and corresponding UV–vis spectra of catalysts are shown in Figures S9–S11 (Supporting Information) at different applied potentials. For the Ag/Co_3_O_4_ NWs and i‐Ag/Co_3_O_4_ NWs, the *j‐t* curves demonstrated that the trend initially strengthens and then weakens within the range of ‒0.25–‒0.45 V versus RHE. We speculate that the heightened current density can be attributed to a phase transition (discussed in the in situ Raman section). At < ‒0.25 V versus RHE, the Ag/Co_3_O_4_ NWs and i‐Ag/Co_3_O_4_ NWs enhanced ammonia production efficiency, leading to the accumulation of NH_3_ on the electrode surface, which impedes the reduction of NO_3_‾, ultimately causing a decline in current density.^[^ ^53^ ^]^ For the Ag NWs, which experience no phase change and maintain low ammonia production efficiency across the entire test potential range, the current density exhibits a stable change trend.

As observed in Figure 2c, the NH_3_ yield rate of i‐Ag/Co_3_O_4_ NWs is increasing with the applied potential negative moving. The FEs for synthesizing NH_3_ reached a peak of 95.8% at −0.35 V and the corresponding NH_3_ yield rate is 314.2 µmol h^−1^ cm^−2^. Impressive, the FEs and NH_3_ yield rate of i‐Ag/Co_3_O_4_ NWs compared favorably with the other reported catalysts (Figure S12 and Table S1, Supporting Information). Ag/Co_3_O_4_ NWs provide a lower NH_3_ yield rate (267.6 µmol h^−1^ cm^−2^) and lower FEs (87.7%), while the Ag NWs have not yet significantly yielded NH_3_ at −0.35 V. Figure 2d shows the Ag NWs exhibit the high NO_2_‾ yield rate and FEs at potentials between ‒0.15 and ‒0.45 V. The NO_2_‾ yield rate of Ag NWs reached a peak of 226.3 µmol h^−1^ cm^−2^ at ‒0.35 V, corresponding to a FE of 60.6%. The NO_3_RR performance studies show that Ag NWs can effectively convert NO_3_‾ to NO_2_‾ but are incapable of converting NO_2_‾ to NH_3_ at low over‐potentials (>‒0.35 V). Since the shell‐layer Co‐based phases of Ag/Co_3_O_4_ NWs and i‐Ag/Co_3_O_4_ NWs can effectively catalyze NO_2_‾ reduction to NH_3_, almost no NO_2_‾ was probed in the electrolyte at <‒0.15 V. In addition, from Figure 2e, the i‐Ag/Co_3_O_4_ NWs attained the highest partial current density of NH_3_ (53.12 mA cm^−2^) and the lowest partial current density of all by‐products (<2.64 mA cm^−2^) at ‒0.25 V versus RHE, suggesting that i‐Ag/Co_3_O_4_ NWs accomplish more effective NH_3_ synthesis.

Control experiments were implemented to confirm that produced NH_3_ is from the NO_3_‾ reduction on i‐Ag/Co_3_O_4_ NWs. As shown in Figure S13 (Supporting Information), the i‐Ag/Co_3_O_4_ NWs still have considerable NH_3_ yield rate and FEs at low concentration of NO_3_‾, but the neglectable NH_3_ yield rate (<0.196 µmol h^−1^ cm^−2^) is measured without NO_3_‾. Moreover, the isotope tracing experiment used ^15^NO_3_‾ as the reactant was performed. As displayed in the ^1^H nuclear magnetic resonance (NMR) spectra (Figure 2f), the ^15^NH_3_ produced by using ^15^NO_3_‾ manifested two peaks, whereas ^14^NH_3_ from ^14^NO_3_‾ showed three peaks.^[^ ^54^ ^]^ All the above results demonstrated that the NH_3_ was delivered from the NO_3_‾ rather than other impurities. To investigate the catalyzed durability of i‐Ag/Co_3_O_4_ NWs for the NO_3_RR, consecutive electrolysis cycles tests were carried out at ‒0.25 V versus RHE. As shown in Figure 2g, the *i*‐*t* curve and the FEs show a little change trend after replacing with a new electrolyte solution each hour. Furthermore, the NH_3_ yield rate in each cycle only fluctuates negligibly (Figure S14a, Supporting Information). The high NH_3_ yield rate of 246.2 µmol h^−1^ cm^−2^ and FEs of 92.3% are held after 10 cycles. As shown in Figure S14b (Supporting Information), the Raman spectra indicate the persistence of solely Co_3_O_4_ and CoOOH components within the Co‐based phase of i‐Ag/Co_3_O_4_ NWs after 10 cycles. The XPS studies reveal minimal alteration in the XPS signals of Ag and Co, suggesting Ag and Co can maintain a stable valence state in the cycle test (Figure S14c,d, Supporting Information). Meanwhile, the morphology of the i‐Ag/Co_3_O_4_ NWs is still maintained post‐electrolysis (Figure S14e, Supporting Information). These results evidence the high stability of i‐Ag/Co_3_O_4_ NWs for NO_3_RR.

**Figure 3:**
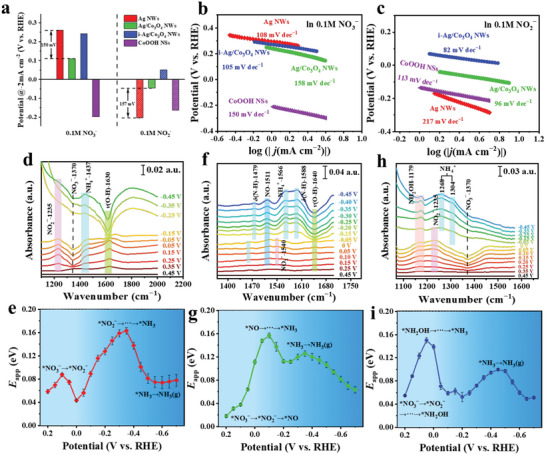
Evaluation of the reaction kinetics and mechanisms of NO_3_RR on the as‐synthesized catalysis. a) The LSV‐derived potentials at a current density of −2 mA cm^−2^ for NO_3_^−^ and NO_2_^−^ reduction on Ag NWs, Ag/Co_3_O_4_ NWs, i‐Ag/Co_3_O_4_ NWs and CoOOH NSs catalysts. The LSV‐derived Tafel slopes of Ag NWs, Ag/Co_3_O_4_ NWs, i‐Ag/Co_3_O_4_ NWs, and CoOOH NSs in b) 0.1 m NO_3_^−^ and c) 0.1 m NO_2_^−^ at pH = 14, respectively. Electrochemical in situ FTIR spectra of d) Ag NWs, f) Ag/Co_3_O_4_ NWs, and h) i‐Ag/Co_3_O_4_ NWs at different potentials in 1 m KOH and 0.1 m NO_3_^−^ solutions. e) Ag NWs, g) Ag/Co_3_O_4_ NWs, and i) i‐Ag/Co_3_O_4_ NWs catalyzed the activation energy for the NO_3_RR at various potentials.

To better understand the active phases involved in the synergistic catalysis of NO_3_RR tandem reaction by i‐Ag/Co_3_O_4_ nanowires. The potential at ‒2 mA·cm^−2^ and Tafel slop are extracted from the LSV curves of the as‐synthesized catalysts in 0.1 m NO_3_‾ and NO_2_‾, respectively (Figure S15, Supporting Information). As shown in **Figure** **3**a–c, Ag NWs show a most positive potential (0.26 V vs RHE) for NO_3_‾ reduction, substantiating the lowest energy barrier of NO_3_‾ reduction on the Ag surface. The corresponding Tafel slope (108 mV dec^−1^) is slightly downward than 120 mV dec^−1^, suggesting that the rate‐determining step (RDS) is the first one‐electron transfer occurring during the NO_3_‾‐to‐NO_2_‾ conversion.^[^ ^55^, ^56^ ^]^ For the core‐shell Ag/Co_3_O_4_ NWs, the potential for NO_3_‾ reduction is negatively moved to 0.11 V versus RHE due to the Ag core covered by the Co_3_O_4_ shell, indicating the energy barrier of NO_3_‾ reduction is increased. The much higher Tafel slope of Ag/Co_3_O_4_ NWs (158 mV dec^−1^) demonstrates that the initial adsorption and activation of NO_3_‾ limits the NO_3_RR process.^[^ ^55^ ^]^ After electrochemical activation treatment, the potential of i‐Ag/Co_3_O_4_ NWs for NO_3_‾ reduction moves to 0.242 V versus RHE and the Tafel slope decreases to 105 mV dec^−1^ close to Ag NWs, indicating the catalytic performance of NO_3_‾ reduction has been enhanced. In comparison to Ag/Co_3_O_4_ NWs, i‐Ag/Co_3_O_4_ NWs exhibit both the phenomenon of Ag core migration and the presence of a new phase of CoOOH. To identify the factors contributing to the enhanced catalytic performance of i‐Ag/Co_3_O_4_ NWs in NO_3_‾ reduction, CoOOH nanosheets were prepared (Figure S16, Supporting Information) and its Tafel slope was analyzed in a 0.1 m NO_3_‾ solution. As shown in Figure 3b, CoOOH showed a large Tafel slope (150 mV dec^−1^) for NO_3_‾ reduction, indicating that the NO_3_‾ reduction on CoOOH is a slow kinetic process. Thus, we decided the enhanced catalytic activity of i‐Ag/Co_3_O_4_ NWs for the NO_3_‾ reduction is the outward migration of Ag atoms rather than the formation of a new phase of CoOOH.

For the NO_2_‾ reduction, Ag NWs exhibit the most negative potential (‒0.204 V vs RHE) and the highest Tafel slope (217 mV dec^−1^), implying a high energy barrier and sluggish kinetics for NO_2_‾ reduction on the Ag surface. In contrast, the potential of Ag/Co_3_O_4_ NWs is positively moving to ‒0.047 V versus RHE, with the corresponding Tafel slope decreasing to 96 mV dec^−1^. After electrochemical activation treatment, the lowest potential (0.05 V vs RHE) and Tafel slope (82 mV dec^−1^) of NO_2_‾ reduction are obtained on the i‐Ag/Co_3_O_4_ NWs. Brunauer–Emmett–Teller (BET) analysis revealed surface areas of 3.08 and 7.65 m^2^ g^−1^ for the Ag/Co_3_O_4_ NWs and i‐Ag/Co_3_O_4_ NWs, respectively (Figure S17, Supporting Information). Obviously, electrochemical activation treatment notably augmented the surface area of i‐Ag/Co_3_O_4_ NWs. Alongside the surface area enhancement, the electrochemical activation treatment also triggered the emergence of novel CoOOH phases. However, CoOOH exhibits a more negative potential (‒0.163V vs RHE) and higher Tafel slope (113 mV dec^−1^) than Ag/Co_3_O_4_ NWs (**‒**0.047 V vs RHE, 96 mV dec^−1^), implying a high energy barrier and sluggish kinetics for NO_2_‾ reduction on the CoOOH surface. Thus, we deduced that the enhanced NO_2_‾ reduction catalyzed by i‐Ag/Co_3_O_4_ NWs, in comparison to Ag/Co_3_O_4_ NWs, primarily resulted from the increased surface area rather than the formation of new CoOOH phases.

Reaction intermediates on the as‐synthesized catalysts were followed by in situ Fourier transforms infrared (FTIR) to identify the mechanism of the NO_3_RR and the role of CoOOH. Figure 3d displays the potential‐dependent FTIR spectra for NO_3_RR over Ag NWs. The in situ FTIR spectra show that a negative band at 1370 cm^−1^ associated with the consumption of NO_3_‾ was observed at 0.25 V versus RHE.^[^ ^57^ ^]^ Simultaneously, an upbeat band at 1235 cm^−1^ can be seen related to the formation of NO_2_‾.^[^ ^58^, ^59^ ^]^ The NO_2_‾‐related band gradually strengthened with the potential negative shift and began to weaken when the potential was negative to **‒**0.25 V versus RHE. Meanwhile, a weak band at 1437 cm^−1^ attributed to NH_4_^+^ appeared, which was apparent at **‒**0.45 V versus RHE. These results prove that the NO_3_‾ first reduced to NO_2_‾ on Ag NWs at 0.25–**‒**0.25 V versus RHE and then, through the successive reaction of NO_2_‾, transformed into NH_3_ when the potential was negative to **‒**0.25 V versus RHE. The activation energy (*E*_a_) may fundamentally represent the NO_3_RR kinetics at each step. To this end, we further conducted the temperature‐dependent kinetic analysis of the Ag NWs catalyst to extract the *E*_a_ of the NO_3_RR process (Figures S19 and S20, Supporting Information). Overall, the resulting *E*_a_ (Figure 3e) shows a low energy barrier of 0.087 eV at 0.1 V versus RHE (corresponding to the reduction of NO_3_‾ to NO_2_‾) and a high energy barrier of 0.163 eV at **‒**0.35 V versus RHE (corresponding to the conversion from NO_2_‾ to NH_3_). The *E*_a_ studies demonstrate that the initial reduction of NO_3_‾ to NO_2_‾ on Ag is a relatively straightforward process. In contrast, the subsequent sequential NO_2_‾ to NH_3_ reactions are particularly slow and represent the RDS for the NO_3_RR process. In addition, the *E*_a_ studies also reveal the reason for prominent NH_3_ production when the potential is negative to **‒**0.45 V versus RHE, because the energy barrier for NH_3_ production has been crossed at **‒**0.45 V versus RHE.

For the core‐shell Ag/Co_3_O_4_ NWs, the in situ FTIR spectra (Figure 3f) showed the fragile band at 1540 cm^−1^ associated with NO_2_‾ formation. In addition, along with the appearance of NO_2_‾ peaks, a positive band at 1511 cm^−1^ related to the NO formation was also observed.^[^ ^60^, ^61^ ^]^ These results indicate that the Co_3_O_4_ shell can effectively catalyze the NO_2_‾ to NO conversion but has deficient catalytic activity for converting NO_3_‾ to NO_2_‾. It is worth pointing out that the band related to NO production increases monotonically over the whole range of test potential, indicating that the NO production rate is more significant than its consumption rate. It also proves that the outer‐layer Co_3_O_4_ phases preferentially catalyze NO_2_‾ to NO conversion rather than catalyze NO to NH_3_ conversion. As the potential negative moves to **‒**0.15 V, the bands at 1566 cm^−1^ and 1588 cm^−1^ attributed to NH_4_^+^ appeared^[^ ^62^, ^63^ ^]^ and strengthened with the potential negative shift. The in situ FTIR demonstrated the sequential NO_3_‾‐NO_2_‾‐NO reactions occur on Ag/Co_3_O_4_ NWs at 0.25–**‒**0.15 V versus RHE and then convert NO into NH_3_ when the potential was negative to **‒**0.15 V versus RHE. The corresponding *E*_a_ (Figure 3g) shows a high energy barrier of 0.157 eV at **‒**0.1 V versus RHE (corresponding to the NO reduction to NH_3_). The other low energy barrier of 0.126 eV at **‒**0.35 V versus RHE may originate from the desorption of NH_3_ from the electrode surface, which is an endothermic process.^[^ ^64^ ^]^ The in situ FTIR and *E*_a_ study fully prove that Co_3_O_4_ can effectively catalyze the conversion of NO_2_‾ to NO in the NO_3_RR process but shows inadequate catalytic activity for NO_3_‾ to NO_2_‾ conversion and NO to NH_3_ conversion.

For the i‐Ag/Co_3_O_4_ NWs, because the inner Ag phases migrated to the catalyst's surface and formed an atomic‐scale Ag/Co interface, the catalytic activity for NO_3_‾ to NO_2_‾ conversion was similar to that of Ag NWs. Figure 3h exhibits the potential‐dependent FTIR spectra for NO_3_RR on i‐Ag/Co_3_O_4_ NWs. As shown in Figure 3h, a robust negative band at 1370 cm^−1^ associated with the consumption of NO_3_‾ and a strong upbeat band at 1235 cm^−1^ related to the formation of NO_2_‾ were simultaneously observed at 0.25 V versus RHE. Interestingly, the band observed at 1179 cm^−1^ indicates the production of NH_2_OH at 0.25 V versus RHE.^[^ ^62^, ^65^ ^]^ By contrast, the spectra recorded with i‐Ag/Co_3_O_4_ NWs do not show the band at 1511 cm^−1^ associated with NO formation. The results showed that intermediate NO was rapidly converted to NH_2_OH.

Compared with Ag/Co_3_O_4_ NWs, a new phase of CoOOH formed in i‐Ag/Co_3_O_4_ NWs after electrochemical activation. The in situ FTIR spectra and the *E*_a_ of CoOOH were measured to investigate its role in the NO_3_RR process. The in situ FTIR spectra show that NH_2_OH formation‐related band appeared at ‒0.15 V versus RHE, the only monitored intermediate associated with the NO_3_RR (Figure S21a, Supporting Information). In addition, the NH_2_OH‐related band increases monotonically over the whole range of test potential, indicating that the NH_2_OH production rate is more significant than its consumption rate. However, the conversion of NH_2_OH to NH_3_ reaction occurs at a potential negative to −0.25 V versus RHE. The corresponding *E*_a_ shows a high energy barrier of 0.234 eV at **‒**0.25 V versus RHE (Figure S21b, Supporting Information). These findings demonstrate that CoOOH has the propensity to produce NH_2_OH, and the subsequent conversion of NH_2_OH into NH_3_ emerges as the RDS of the NO_3_RR process. Therefore, the rapid transformation of NO to NH_2_OH catalyzed by i‐Ag/Co_3_O_4_ NWs can be attributed to the CoOOH formation. The CoOOH exhibits a more negative potential (‒0.25 V vs RHE) than i‐Ag/Co_3_O_4_ NWs (0.25 V vs RHE) for NH_2_OH formation, which is caused by the isolated CoOOH phase lacks synergy from Ag and Co_3_O_4_.

As the potential negative moves to **‒**0.05 V, the bands at 1268 and 1304 cm^−1^ attributed to NH_4_^+^ appeared^[^ ^63^ ^]^ and strengthened with the potential negative shift. The in situ FTIR demonstrated the sequential NO_3_‾‐NO_2_‾‐NO‐NH_2_OH reactions occur on i‐Ag/Co_3_O_4_ NWs at 0.25–‒0.05 V versus RHE and then convert NH_2_OH into NH_3_ when the potential was negative to ‒0.05 V versus RHE. Similar to Ag/Co_3_O_4_ NWs, the resulting *E*_a_ (Figure 3i) shows a high energy barrier of 0.151 eV at 0.05 V versus RHE (corresponding to the conversion from NH_2_OH to NH_3_) and a low energy barrier of 0.099 eV at ‒0.45 V versus RHE (corresponding to the desorption of NH_3_). The *E*_a_ studies demonstrate the relatively easy sequential NO_3_‾‐NO_2_‾‐NO‐NH_2_OH reactions occurring on i‐Ag/Co_3_O_4_ NWs. In contrast, transforming NH_2_OH into the final product is the RDS for the NO_3_RR process, which aligns with the RDS in NO_3_RR catalyzed by CoOOH. Additionally, it is noted that the onset potential for the mass production of NH_3_ is **‒**0.45, **‒**0.15, and **‒**0.05 V versus RHE on Ag NWs, Ag/Co_3_O_4_ NWs, and i‐Ag/Co_3_O_4_ NWs, respectively. These findings further suggest the synergy of the Ag (catalyze NO_3_‾ to NO_2_‾ conversion), Co_3_O_4_ (catalyze NO_2_‾ to NO conversion), and CoOOH (catalyze NO to NH_2_OH conversion) phases in i‐Ag/Co_3_O_4_ NWs can efficiently convert NO_3_‾ to NH_3_ at low potential, thus effectively reducing the energy consumption in the NO_3_RR process.

**Figure 4:**
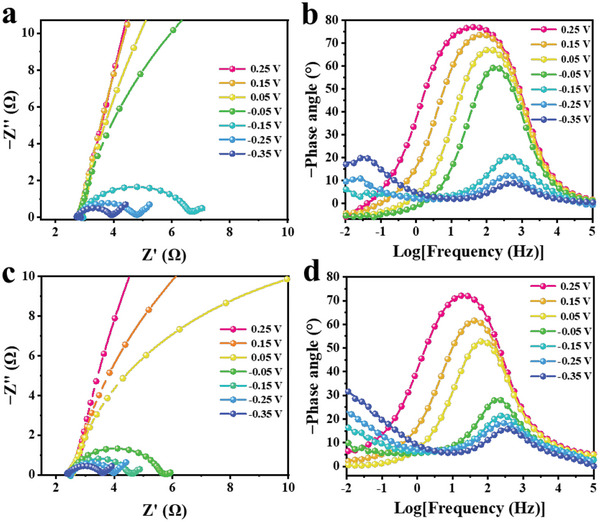
Operando EIS measurements of the NO_3_RR process. Nyquist plots for a) Ag/Co_3_O_4_ NWs and c) i‐Ag/Co_3_O_4_ NWs at different applied potentials in 0.1 m NO_3_^−^ and 1 m KOH. The corresponding Bode phase plots of b) Ag/Co_3_O_4_ NWs and d) i‐Ag/Co_3_O_4_ NWs at different applied potentials.

Electrochemical impedance spectroscopy (EIS) is a potentially helpful experimental tool for probing the kinetics of electrocatalytic reactions and the properties of the electrode/electrolyte interfaces.^[^ ^66^, ^67^ ^]^ To this end, operando EIS measurements were performed to deeply understand reaction kinetics in 1 m KOH and 0.1 m KNO_3_ solution. **Figure** **4**a,c illustrated the Nyquist plots of the measured impedance of the NO_3_RR process on Ag/Co_3_O_4_ NWs and i‐Ag/Co_3_O_4_ NWs at the potential range of 0.25 to **‒**0.35 V versus RHE. An equivalent circuit was employed to fit these Nyquist plots at various applied potentials (Figure S18, Supporting Information). The equivalent circuit consists of the solution resistance (*R*_s_), constant phase element (CPE), and charge‐transfer resistance (*R*_ct_). The results from the EIS fitting are listed in Table S2 (Supporting Information). The adsorption behavior of the reactants (intermediates) on the catalyst surface can be reflected by *R*_ct_ and CPE. Similar studies have been reported in the HER and OER processes.^[^ ^68^, ^69^, ^70^, ^71^ ^]^ Since these two catalysts possess similar *R*_s_, the variation of total resistance (*R*_total_) was determined by *R*_ct_. At each applied potential, the *R*_total_ of i‐Ag/Co_3_O_4_ NWs is smaller than that of Ag/Co_3_O_4_ NWs, suggesting a faster electron transfer and faster kinetics in adsorbed reactants (intermediates) during NO_3_RR are realized in i‐Ag/Co_3_O_4_ NWs.^[^ ^70^, ^71^ ^]^

The Bode phase plot of Ag/Co_3_O_4_ NWs and i‐Ag/Co_3_O_4_ NWs at each applied potential is shown in Figure 4b,d. The impendence response at the higher frequency region (10^0^–10^4^ Hz) could correlate to the NO_3_RR reactions over the as‐synthesized catalysts. The phase peak for i‐Ag/Co_3_O_4_ NWs at the fixed potential showed a lower phase angle and shift than the Ag/Co_3_O_4_ NWs, indicating the faster kinetic rate of intermedia reactions during the NO_3_RR process.^[^ ^71^ ^]^ Combined with the in situ FTIR studies, the phase peak change for Ag/Co_3_O_4_ NWs at the potential range of ‒0.05–‒0.15 V versus RHE is attributed to the NH_3_ formation. In contrast, the corresponding phase peak change of i‐Ag/Co_3_O_4_ NWs at 0.05–**‒**0.05 V versus RHE, moved 100 mV positively compared with the Ag/Co_3_O_4_ NWs. In addition, the phase peak for the Ag/Co_3_O_4_ NWs at the lower frequency region (10^−2^–10^0^ Hz) can be observed when applied potentials reached **‒**0.15 V versus RHE, the corresponding Nyquist plots appear Warburg line, suggesting the kinetic rate of yield NH_3_ is fast and the electrode reaction is limited by diffusion at **‒**0.15 V versus RHE.^[^ ^68^, ^72^ ^]^ The corresponding phase peak and Warburg lines for i‐Ag/Co_3_O_4_ NWs are found at **‒**0.05 V versus RHE. These results demonstrate that the i‐Ag/Co_3_O_4_ NWs can further expedite the NO_3_RR reaction and reduce the overpotential for NH_3_ formation.

To identify the active phases for NO_3_RR, the in situ Raman spectra of the three catalysts were measured under a series of applied potentials in 1 m KOH and 0.1 m KNO_3_ solution. Figure S24 (Supporting Information) shows the Raman spectra of Ag NWs at reducing potentials related to NO_3_RR. The dominant bands located at 692, 749, 832, 943,1062, 1142, and 1267 cm^−1^ are ascribed to the N─C═O bend vibration, the symmetric stretch vibration of heterocyclic C─N─C, the in‐plane pyrrolidinone ring breathing, C─C in‐plane bending, the stretch vibration of C─N, the weak ring CH_2_ twist, and the in‐plane C─H of PVP, respectively.^[^ ^73^, ^74^, ^75^ ^]^ The distinct bands of PVP are derived from the absorption of the introduced PVP onto the [100] facet of Ag NWs during the synthesis process.^[^ ^76^ ^]^ The reason for the attenuation of Raman peaks is that the PVP ligand will desorption from Ag NWs surface at a more negative potential.^[^ ^77^ ^]^ The characteristic Raman peaks of high‐valent Ag were not observed, indicating that the active phase of Ag NWs for reducing NO_3_‾ to NH_3_ is related to metallic Ag.

**Figure 5:**
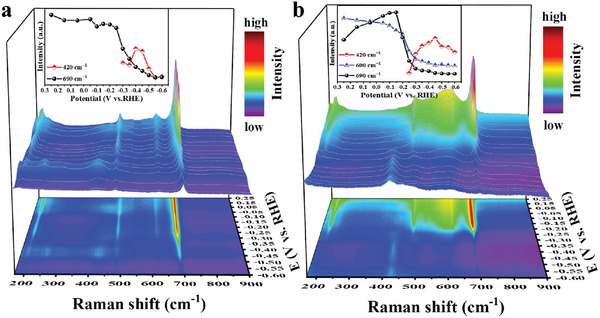
In situ Raman spectra of the catalysts. In situ Raman spectra of a) Ag/Co_3_O_4_ NWs and b) i‐Ag/Co_3_O_4_ NWs at different applied potentials in electrolytes containing 0.1 m NO_3_^−^ and 1 m KOH. The inset figure is the relative change tendency of the Raman peak at 420, 600, and 690 cm^−1^ as a function of applied potential.

On the Ag/Co_3_O_4_ NWs catalysts, the initial broad bands at 690, 620, 521, 480, and 195 cm^−1^ associated with Co_3_O_4_ phases, persist as low as **‒**0.25 V versus RHE (**Figure** **5**a). Remarkably, at <**‒**0.25 V versus RHE, the characteristic Raman peaks of Co_3_O_4_ are fast attenuated and a peak emerges at 420 cm^−1^ assigned to Co(OH)_2_,^[^ ^23^ ^]^ suggesting the gradual conversion of Co_3_O_4_ into Co(OH)_2_. The most substantial Raman peak of Co(OH)_2_ is obtained at **‒**0.45 V versus RHE and then decreases with moving potentials negatively. This result indicates the gradual conversion of Co(OH)_2_ into metallic Co at <**‒**0.45 V versus RHE. Based on these results, we can conclude the active phase of Ag/Co_3_O_4_ NWs for NO_3_RR is related to Co_3_O_4_ at > **‒**0.25 V versus RHE, while that is Co(OH)_2_ at **‒**0.25–**‒**0.45 V versus RHE, and metallic Co at <**‒**0.45 V versus RHE.

On the i‐Ag/Co_3_O_4_ NWs catalysts, the Raman peaks associated with Co_3_O_4_ and CoOOH phases quickly decayed at a potential range from ‒0.15 to ‒0.30 V versus RHE, while a Raman peak at 420 cm^−1^ assigned to Co(OH)_2_ emerged at ‒0.25 V versus RHE. Although the Raman peaks of the Co(OH)_2_ phase persist to potentials as low as ‒0.60 V versus RHE, they began to decrease gradually at < ‒0.45 V versus RHE (Figure 5b). These results suggest the phase evolution of the i‐Ag/Co_3_O_4_ NWs similar to Ag/Co_3_O_4_ NWs at a relatively negative potential. Namely, the Co^3+/2+^‐containing Co_3_O_4_ and CoOOH are first reduced to Co^2+^‐dominated Co(OH)_2_ and then to metallic Co. Thus, the active phase of i‐Ag/Co_3_O_4_ NWs for NO_3_RR is also the Co(OH)_2_ or metallic Co phases at < **‒**0.30 V versus RHE. The difference is that the i‐Ag/Co_3_O_4_ NWs contain two active phases of Co_3_O_4_ and CoOOH for catalyzing NO_3_RR at > **‒**0.20 V versus RHE. As shown in the inset of Figure 5b, the Raman band at 600 cm^−1^ attributes to the symmetric stretching mode of the CoO_6_ octahedra in CoOOH^[^ ^78^ ^]^ gradual decay with decreasing potentials from 0.25 to **‒**0.20 V versus RHE. Meanwhile, the band at 690 cm^−1^ assigned to the symmetric stretching mode of the CoO_6_ octahedral unit in Co_3_O_4_ gets stronger.^[^ ^79^ ^]^ Based on this result, we speculate that the surface of CoOOH would lose some H atoms under the NO_3_RR reaction process and transform its CoO_6_ octahedral mode to that of Co_3_O_4_, the similar deprotonation of cobalt hydroxide/oxyhydroxide has been reported in the HER and OER study.^[^ ^39^, ^48^ ^]^ This finding reveals the reason for rapidly producing NH_2_OH over the i‐Ag/Co_3_O_4_ NWs catalyst (Figure 3h). This is because the CoOOH can provide the extra H atoms, dramatically accelerating NO's hydrogenation step (Figure S25, Supporting Information).

The in situ Raman spectra unveil the emergence of the Co(OH)_2_ phase within both Ag/Co_3_O_4_ NWs and i‐Ag/Co_3_O_4_ NWs when the potential drops below ‒0.25 V versus RHE. Thus, the in situ FTIR spectra and the *E*_a_ of Co(OH)_2_ at various potentials were measured to investigate its role in the NO_3_RR process. Furthermore, the overpotential and Tafel slope of NO_3_‾/NO_2_‾ reduction catalyzed by Co(OH)_2_ were re‐analyzed to evaluate the catalytic activity of Co(OH)_2_. Figure S26a (Supporting Information) shows five evident absorption bands in the FTIR spectra of Co(OH)_2_. 1) At 0.25 V versus RHE, an upward band attributed to NO_3_‾ appears at 1373 cm,^−1[^ ^57^ ^]^ indicating adsorption of NO_3_‾ on the Co(OH)_2_ surface; 2) At **‒**0.25 V versus RHE, the upbeat bands at 1155 and 1267/1437 cm^−1^ were ascribed respectively to NH_2_ and NH_4_,^+[^ ^63^ ^]^ indicating the formation of NH_2_ and NH_4_^+^ species on the Co(OH)_2_ surface; 3) at the same time, the upward band at 1373 cm^−1^ switched downward, indicating NO_3_‾ is rapidly consumed and reduced into NH_2_ and NH_4_^+[^ ^57^ ^]^; 4) The upward band ≈1670 cm^−1^ was attributed to water electrolysis responsible of hydrogen generation involved in the hydrodeoxidation of NO_3_‾.^[^ ^52^ ^]^ In situ FTIR study revealed that due to the absence of synergistic action among Ag, Co_3_O_4_, and CoOOH, Co(OH)_2_ exhibited limited efficacy in catalyzing the reduction of NO_3_‾ to NO_2_‾ and subsequent conversion to NH_3_ when the working potential greater than **‒**0.25 V versus RHE. This conclusion is further supported by the large overpotential and Tafel slope observed during the reduction of NO_3_‾, and NO_2_‾ catalyzed by Co(OH)_2_ (FigureS27, Supporting Information). At <**‒**0.25 V versus RHE, the energy barrier for NH_3_ production has been crossed (Figure S26b, Supporting Information). Co(OH)_2_ can rapidly catalyze the conversion of NO_3_‾ into NH_3_ via NH_2_ intermediates. Hence, we conclude that the in situ generation of Co(OH)_2_ at potentials below **‒**0.25 V versus RHE could facilitate the catalytic conversion of NH_2_OH into NH_2_, thereby promoting the NH_3_ synthesis.

**Figure 6:**
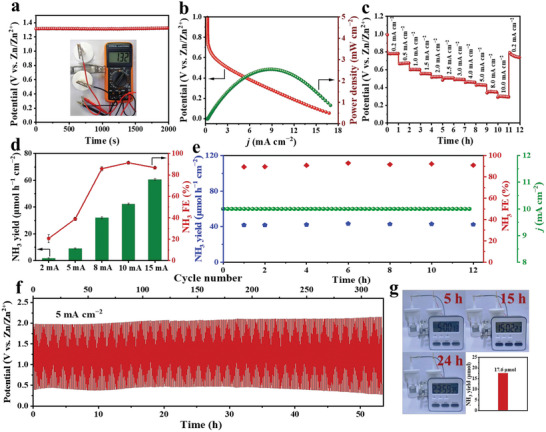
The electrochemical performance of Zn**–**NO_3_^−^ battery. a) Open circuit voltage of i‐Ag/Co_3_O_4_ NWs‐based Zn–NO_3_^−^ battery. b) The discharging curve and the resultant power density curve of the i‐Ag/Co_3_O_4_ NWs‐based Zn–NO_3_^−^ battery. c) Discharging curves at different current densities. d) FE and NH_3_ yield rate of Zn–NO_3_^−^ battery with i‐Ag/Co_3_O_4_ NWs catalyst cathode. e) The long‐term NO_3_RR experiment and the corresponding NH_3_ FE and yield with the Zn–nitrate battery system. f) Discharge–charge processes of Zn–NO_3_^−^ battery at a constant current density of 5 mA cm^−2^. g) A photograph of the Zn–NO_3_^−^ battery powering an electronic timer to work for 24 h and yield NH_3_ of 17.60 µmol.

Aqueous zinc‐nitrate batteries offer an attractive opportunity to convert NO_3_‾ into NH_3_ and supply electric energy concurrently. Therefore, we construct a battery by anchoring the i‐Ag/Co_3_O_4_ NWs on carbon paper as the cathode and Zn plate as the anode. As shown in **Figure** **6**a, i‐Ag/Co_3_O_4_ NWs‐based battery exhibits a stable open circuit potential of 1.32 V versus Zn/Zn^2+^, higher than Ag/Co_3_O_4_ NWs‐based battery (1.23 V vs Zn/Zn^2+^) in Figure S28a (Supporting Information) and superior to most Zn‐NO_3_‾ batteries have been reported.^[^ ^80^ ^]^ Figure 6b shows the discharge curves of the i‐Ag/Co_3_O_4_ NWs‐based Zn‐NO_3_‾ battery. The discharging curve for such Zn‐NO_3_‾ cell shows an increased output current density with a more negative cathodic potential. The power density of the Zn‐NO_3_‾ cell reaches the peak of 2.56 mW cm^−2^, higher than for Ag/Co_3_O_4_ NWs‐based Zn‐NO_3_‾ battery (0.94 mW cm^−2^, Figure S28b, Supporting Information). Figure 6c shows the discharge curve of the Zn‐NO_3_‾ battery under different current densities from 0.2 to 10 mA cm^−2^. The voltage initially levels off at 0.78 V and stays stable for 1 h. The other steps display the same stability, suggesting excellent mass transfer and conductivity. Figure 6d shows the NH_3_ yield and corresponding FE when discharging with different output current densities. The i‐Ag/Co_3_O_4_ NWs‐based Zn‐NO_3_‾ battery delivers a high NH_3_ yield rate of 42.70 µmol h^−1^ cm^−2^ and a high FE of 91.4% at 10 mA cm^−2^. In addition, the FE holds at 91.0% and the NH_3_ yield rate of 42.40 µmol cm^−2^ h^−1^ is attained after consecutive discharging measurements for 12 h at 10 mA cm^−2^ (Figure 6e), verifying the long‐time durability of i‐Ag/Co_3_O_4_ NWs‐based Zn‐NO_3_‾ battery. Figure 6f shows the discharge–charge processes of the Zn‐NO_3_‾ battery at a constant current density of 5 mA cm^−2^. Such a Zn‐NO_3_‾ battery exhibits a stable charging and discharging platform for 53 h (320 cycles). Moreover, i‐Ag/Co_3_O_4_ NWs‐based Zn‐NO_3_‾ battery can power an electronic timer for >24 h and yield NH_3_ of 17.6 µmol (Figure 6g). Thus, the i‐Ag/Co_3_O_4_ NWs‐based Zn‐NO_3_‾ battery achieves bifunctional ability for NO_3_‾ to NH_3_ conversion and produces electric energy, broadening the Zn‐based batteries field.

## 3 Conclusion 

In summary, we achieved the efficient reduction of NO_3_‾ to NH_3_ in the alkaline electrolyte by designing an Ag/Co_3_O_4_/CoOOH NWs (i‐Ag/Co_3_O_4_ NWs) tandem catalyst, which is ascribed to the synergistic action of both active phases in the catalyst. In this tandem catalysis system, NO_3_‾ ions are reduced to NO_2_‾ preferentially on Ag phases, and then the NO_2_‾ intermediates are converted to NO on Co_3_O_4_ phases. The CoOOH phases benefit the hydrogenation step of NO and can effectively catalyze NO reduction to NH_2_OH and then to NH_3_ due to the CoOOH providing the extra H atoms during the NO_3_RR reaction. The in situ FTIR and *E*_a_ studies demonstrate that the conversion of NH_2_OH into NH_3_ is the potential RDS with an uphill energy change of 0.151 eV over i‐Ag/Co_3_O_4_ NWs. A high‐rate NH_3_ generation at low overpotentials was achieved by working in tandem. The i‐Ag/Co_3_O_4_ NWs tandem catalysts show an excellent FE for NH_3_ (94.3%) and super‐high NH_3_ yield rate of 253.7 µmol h^−1^ cm^−2^ in 1 m KOH and 0.1 m KNO_3_ solution at **‒**0.25 V versus RHE. Furthermore, the i‐Ag/Co_3_O_4_ NWs‐based Zn‐NO_3_‾ battery is constructed, and the bifunctional ability for NO_3_‾ to NH_3_ conversion and produce electric energy is achieved. This work highlights the promise of tandem catalysts for NO_3_RR and broadens the field of Zn‐based batteries in the application of electrocatalysis.

## Acknowlegments

This work was supported by the National Science Foundation of China (NSFC nos. 22 105 147, 51 972 238, and U21A2081), and the Wenzhou Science and Technology Bureau (no. 4 051 000).

## Conflict of Interest

The authors declare no conflict of interest.

## Supporting information

Supporting Information

## Data Availability

The data that support the findings of this study are available from the corresponding author upon reasonable request.
