# Supplementary material for: Ag‐Co3O4‐CoOOH‐Nanowires Tandem Catalyst for Efficient Electrocatalytic Conversion of Nitrate to Ammonia at Low Overpotential via Triple Reactions
Source: Adv Sci (Weinh). 2023 Oct 11;10(33):2303789. doi: 10.1002/advs.202303789 (PMC10667848; doi:10.1002/advs.202303789)
Supplement: Supplementary file 1 — Supporting Information [file ADVS-10-2303789-s001.pdf]

ADVANCED SCIENCE
Open Access

Supporting Information

for *Adv. Sci.*, DOI 10.1002/advs.202303789

Ag-Co_3O_4-CoOOH-Nanowires Tandem Catalyst for Efficient Electrocatalytic Conversion of Nitrate to Ammonia at Low Overpotential via Triple Reactions

Shilu Wu, Yingyang Jiang, Wenjie Luo, Peng Xu, Longlong Huang, Yiwen Du, Hui Wang, Xuemei Zhou, Yongjie Ge, Jinjie Qian*, Huagui Nie* and Zhi Yang**

Supporting Information

Ag-Co_3O_4-CoOOH-Nanowires Tandem Catalyst for Efficient Electrocatalytic Conversion of Nitrate to Ammonia at Low Overpotential via Triple Reactions

Shilu Wu, Yingyang Jiang, Wenjie Luo, Peng Xu, Longlong Huang, Yiwen Du, Hui Wang, Xuemei Zhou, Yongjie Ge, Jinjie Qian*, Huagui Nie* and Zhi Yang**

Key Laboratory of Carbon Materials of Zhejiang Province, College of Chemistry and Materials Engineering, Wenzhou University, Wenzhou, 325035, P. R. China.

* E-mail: geyongjie1220@126.com (Y Ge); jinjieqian@wzu.edu.cn (J Qian); huaguinie@126.com (H Nie); yang201079@126.com (Z Yang)

Experimental Section

Materials.

$AgNO_3$ (AR), $CuCl_2$ (AR), PVP (K-30, GR), acetone (AR), ethylene glycol (CP) were purchased from Sinopharm Chemical Reagent Co., Ltd. (Shanghai, China). Cobalt(II) acetate tetrahydrate (AR), Cobalt(II) nitrate hexahydrate (AR), KOH (ACS), oleylamine (AR), n-hexane (GC) were obtained from Aladdin Reagent Co. Ltd.

(Shanghai, China). NaOH (ACS), KNO_3 (ACS), $NaNO_2$ (AR), Salicylic acid (AR), Sulfanilamide (ACS) were obtained from Sigma-Aldrich. All chemicals were used without further purification. Ultrapure water (≥18.2 MΩ cm) purified from a Elix5-Milli-Q ultrapure water system were used throughout the experiments.

Synthesis of Ag/Co_3O_4 nanowires (Ag/Co_3O_4 NWs).

Ag NWs were synthesized through the polyol reduction method reported previously.[1] To obtain Ag/Co_3O_4 NWs, 10 mg Ag NWs were dispersed in 10 mL of 30 mM $Co(OAc)_2 \cdot 4H_2O$ oleylamine solution and heated at 90 °C for 10 min under vigorous stirring in the Ar atmosphere, And then the temperature was raised to 200 °C for the reaction for 1 h. The reaction was quenched by cooling the flask in a room-temperature water bath. The resultant Ag/Co_3O_4 NWs were washed with a mixture of ethanol, n-hexane, and acetone thrice, then stored in ethanol, forming a bulk dispersion (3 mg/mL).

Synthesis of i-Ag/Co_3O_4 nanowires (Ag/Co_3O_4/CoOOH NWs).

The carbon paper (CP) loaded with Ag/Co_3O_4 NWs was used as the working electrode in a typical three-electrode system. Ag/AgCl (saturated KCl) and a platinum mesh were employed as the reference and counter electrodes, respectively. The carbon paper loaded with Ag/Co_3O_4 NWs was polarized by cyclic voltammetry (CV) range from 0.05 to 2.05 V versus RHE in an Ar-saturated 1 M KOH solution for 4 cycles, followed by gentle rinsed with water and acetone and drying in Ar flow.

Synthesis of $Co(OH)_2$ NSs and CoOOH NSs.

The electrochemical deposition was carried out by galvanostatic electrolysis in a two-electrode cell.[2] A carbon paper (2 cm × 1 cm) and a graphite electrode (1.8 cm^2, spectral grade) were used as the working and counter electrodes. $Co(OH)_2$ NSs was electrodeposited on carbon paper in 15 mL of 0.02 M $Co(NO_3)_2$ + 0.1 M NH_4Cl solution at –10 mA cm^{-2} for 20 min. And CoOOH NSs was fabricated by in situ anodic oxidation of $Co(OH)_2$ NSs in a solution of 0.01 M $(NH_4)_2SO_4$ at 2 mA cm^{-2} for 30 min.

Material characterization.

SEM was performed using a Nova Nano SEM 200 (FEI, USA) scanning electron

microscope. TEM images were carried out on an FEI Tecnai G2F 20 TEM system using copper grids (JEOL, Japan). Raman spectra were measured from a Renishaw in Via Raman spectrometer (Renishaw, UK). A 50× long-working distance objective (NA, 0.5) was used to focus the laser beam onto the sample and to collect the Raman signals in the backscattering mode. The 785 nm line from an argon ion laser, with a power of 50 mW was used as the excitation source. Fourier-transform infrared (FT-IR) spectra were measured on an IRTracer-100 spectrometer (Shimadzu, Japan). UV-visible absorption spectra were recorded on a UV-1800 UV-visible spectrophotometer (Shimadzu, Japan). XPS was recorded using an ultrahigh-vacuum setup (SES 2002, Gammadata-Scienta) equipped with a monochromatic Al Kα X-ray source (15 kV, 10 mA emission current). The binding energies were calibrated based on the C 1s feature at 284.8 eV. The nuclear magnetic resonance (NMR) spectroscopy was performed on an AVANCE III AV500 spectrometer.

Electrochemical tests.

The electrocatalytic tests were performed using a typical three-electrode system connected to the CHI 660E electrochemical workstation (CHI Instrument, China) in a typical H-type cell. The catalysts supported by carbon paper, Ag/AgCl (saturated KCl), and platinum mesh were used as the working electrode, reference, and counter electrodes, respectively. The electrolytes were Ar-saturated 1 M KOH containing different NO_3^- or NO_2^- concentrations. The LSV curves were collected at a scan rate of 10 $mV \cdot s^{-1}$. Tafel slopes were extracted from near static LSV. All potentials were calibrated to the RHE reference scale using $E_{RHE} = E_{Ag/AgCl} + 0.204\ V + 0.0591 \times pH$. The current density was normalized to the geometric electrode area (~1 cm^2). Potentiostatic measurements were performed for 1 h in 30 mL cathode electrolyte, and then the electrolyte was stored at 4 °C (no more than 2 days) before analysis. To evaluate the long-term stability of i-Ag/Co_3O_4 NWs for NO_3RR, the electrolyte solution was collected and analyzed for NH_3 production after every hour of electrolysis. To maintain consistency, a fresh electrolyte solution was used for each cycle of one-hour electrolysis. The C_{dl} was determined by CV scanning in a non-faradaic potential

window at different scan rates (10-70 mV·s^{-1}). The plot of the capacitive anode and cathode current differences [($j_a - j_c$)/2] at a set potential against the CV scan rates shows a linear relationship, and the slope is C_{dl}. Electrochemical impedance spectroscopy (EIS) tests were performed using an Autolab potentiostat (Metrohm, Switzerland). EIS was performed at different applied potentials versus RHE in the 10^{-2} - 10^{5} Hz frequency range.

Assembly of the zinc-nitrate battery and electrochemical test.

The CP-supported i-Ag/Co_3O_4 NWs (1×1 cm^2) were employed as the cathode and the Zn plate (1.5×2 cm^2) was used as the anode for the zinc-nitrate battery. A typical H-type cell that contains 25-mL cathode electrolyte (1 M KOH + 0.1 M KNO_3) and 25-mL anode electrolyte (1 M KOH) separated by a bipolar membrane. The discharging polarization curves with a scan rate of 10 mV·s^{-1} and galvanostatic tests were conducted using CHI 660E workstation and Neware test system at room temperature, respectively. After the electrochemical test, the electrolyte was diluted for subsequent detection.

The power density (P) of zinc-nitrate battery was determined by $P = I \times V$, where I and V are the discharge current density and voltage, respectively.

The electrochemical reactions in the Zn-nitrate battery are presented as follows:

Cathode reaction: $NO_3^- + 7H_2O + 8e^- \rightarrow NH_4OH + 9OH^-$

Anode reaction: $4Zn + 8OH^- \rightarrow 4ZnO + 4H_2O + 8e^-$

Overall reaction: $4Zn + NO_3^- + 3H_2O \rightarrow 4ZnO + NH_4OH + OH^-$

Since a bipolar membrane separates the cathode and anode, the electrode potentials can be calculated individually:

$$E_{\text{cathode}} = -\frac{1}{nF}\left(\Delta G_{cat} + RT\ln\frac{[OH^-]^9[NH_4OH]}{[NO_3^-]}\right) = 0.14\ \text{V}$$

$$E_{\text{anode}} = -\frac{1}{nF}\left(\Delta G_{ano} + RT\ln\frac{1}{[OH^-]^2}\right) = -1.25\ \text{V}$$

Where n, F, ΔG, R, and T are electron transfer numbers, the Faraday constant (96485 C mol^{-1}), standard molar Gibbs free energy change of chemical reaction at 298 K, gas constant (8.314 J mol^{-1} K^{-1}), and reaction temperature (298 K), respectively. The concentrations of [OH^-] in the cathode and anode cells are 1 mol/L, respectively. [NO_3^-]

is 0.1 mol/L. Assuming [NH_4OH] is 10^{-3} mol/L in the cathode cell. Therefore, we have

$E_{overall} = E_{cathode} - E_{anode} = 1.39$ V

Determination of ion concentrations.

NH_4^+ quantification.

The produced NH_3 was quantitatively determined by the indophenol blue method.[3,4] Typically, a certain amount of electrolyte was removed from the reaction cell and diluted to 2 mL. Then, 2 mL of 1 M NaOH solution containing citrate dihydrate (5 wt%) and salicylic acid (5 wt%) (stored at 4 °C) and 1 mL of freshly prepared 0.05 M NaClO were added. The resulting mixture was then briefly shaken to ensure proper mixing of the components. Finally, 0.2 mL of 1 wt% sodium nitroferricyanide solution (stored at 4 °C) was added for the color reaction. Following a 2-hour incubation period at room temperature, the resulting solution was measured using an ultraviolet-visible (UV-Vis) spectrophotometer. The absorbance at 655 nm was used to determine the concentration of NH_3. To quantify the amount of NH_3, a calibration curve was built using a standard NH_4Cl solution in 1 M KOH.

NO_2^- quantification.

A specific colour reagent for NO_2^- quantification was prepared by mixing 0.08 g of N-(1-naphthyl) ethylenediamine dihydrochloride, 1.6 g of sulfonamide and 4 mL of phosphoric acid (85 wt%, ρ =1.7 g/mL) with 20 mL of deionized water.[5,6] In a typical colorimetric test, 1 mL HCl (1 M) was firstly added into the 5 mL of diluted post-electrolysis electrolytes, and then 0.1 mL of colour reagent was added and shaken to obtain a uniform solution. The UV-Vis absorbance at 540 nm was recorded after 30 min at room temperature. The amount of NO_2^- was determined using a calibration curve of $NaNO_2$ solutions.

Calculation of the NH_3 yield rate and Faradaic efficiency.

The FE was defined as the charge consumed for forming a specific product (e.g. NH_3) divided by the total charge passing through the electrodes (Q) during electrolysis. Given that eight electrons are consumed to produce one NH_3 molecule, FE_{NH3} and NH_3 yield rate (Y_{NH3}) was calculated according to the following equation:

$$FE_{NH_3} = \frac{8 V_{NH_3} c_{NH_3} F}{Q} \qquad Y_{NH_3} = \frac{V_{NH_3} c_{NH_3}}{At}$$

where F is the Faraday constant (96485 C mol^{-1}), Q is the total charge passing the electrode, c_{NH3} is the molar concentration of detected NH_3, V_{NH3} is the volume of the electrolytes (30 mL), A is the electrode geometric area (1 cm^2), and t is the reaction time.

Given that two electrons are consumed to produce one NO_2^- molecule, the FE of NO_2^- can be calculated as follows:

$$FE_{NO_2^-} = \frac{2 V_{NO_2^-} c_{NO_2^-} F}{Q}$$

where C_{NO2-} is the molar concentration of detected NO_2^-.

The reaction apparent activation energy tests.[7]

To extract the apparent activation energy (E_a) for the NO_3RR, the electrochemical measurements of the catalysts were conducted in 1 M KOH solution containing 0.1 M KNO_3 at different temperatures. For heterogeneous electrocatalytic reactions, the current density can be expressed from E_a according to the following Arrhenius equations.[8]

$$j = A_a \exp(-\frac{E_a}{RT})$$

where A_a is the apparent pre-exponential factor, R is the ideal gas constant (8.314 J·K^{-1}·mol^{-1}), T is the temperature in Kelvin (K). Therefore, E_a can be further calculated by fitting the slope of the Arrhenius plot the following equations.[9]

$$\left| \frac{\partial(\log_{10} j)}{\partial 1/T} \right|_{\eta} = -\frac{E_a}{2.303R}$$

while the intercept of $\log_{10} j$ vs. $1/T$ plot is the logarithm of A_a.

$K^{15}NO_3$ isotope labeling experiments.

The isotope labeling experiment was carried out in 1 M KOH solution containing 0.1 M $K^{15}NO_3$ (98% ^{15}N atom) by chronoamperometry measurements for 1 h at –0.25 V (*vs.* RHE). Briefly, the pH of the processed electrolyte was adjusted to 3 with a 4 M H_2SO_4 solution.[10] Then, 400 µL of electrolyte and 150 µL of deuterium oxide (D_2O) were added into the NMR tube, and $^{15}NH_4^+$ in the electrolyte was detected using ^{1}H

NMR(500 MHz).[11]

In situ FTIR spectroscopy.

FTIR measurements were performed with an IRTracer-100 spectrometer. The electrochemical cell was assembled on top of a CaF_2 prism, and the electrode was situated against this prism to form a thin layer. The measurements were performed under external reflection (**Figure S18**). The electrochemical cell was assembled by a three-electrode configuration with a counter electrode of Pt wire, a reference electrode of Ag/AgCl (saturated KCl), and a working electrode prepared by dropping 20 μL ink of electrocatalyst on an Au sheet. Electrolyte was Ar-saturated 1 M KOH with 0.1M NO_3^-. FTIR spectra were obtained from an average of 512 scans with a resolution of 8 cm^{-1} at the selected potentials. The potentiostatic model is adopted, and the potentials are scanned from 0.35 V to –0.45 V (*vs.* RHE) compared to the reference potential (0.45 V *vs.* RHE). The spectra were reported as $-lg(R/R_0)$, where R is the reflectance at a set potential, and R_0 is the reflectance at the reference potential. Thereby the ratio gives positive bands for species formation at the sample potential, and negative bands correspond to the loss of species at the sample potential.

In situ Raman spectroscopy.

Raman spectroscopy was performed with a Renishaw in Via Raman spectrometer (Renishaw, UK) equipped with a 785 nm laser as the excitation source, a 50× objective, a monochromator (1200 grooves/mm grating). The in situ Raman spectra were collected under controlled potentials. The electrolytic cell was homemade by Teflon with a piece of round quartz glass as a cover to protect the objective (**Figure S23**). The Au electrode modified with the catalyst was used as the working electrode, Pt wire and Ag/AgCl electrode as the counter and reference electrodes, respectively. The surface of the working electrode was positioned 100-200 μm from the glass window. Each spectrum is an average of three continuously acquired spectra with a collection time of 50 s each.

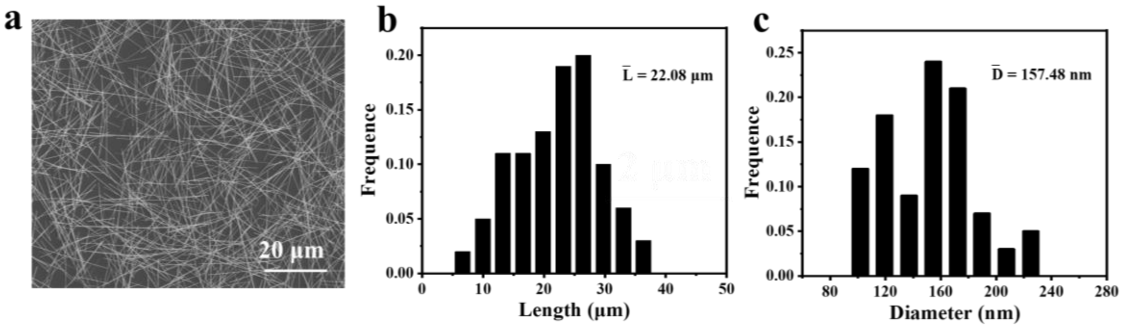

Figure S1. (a) SEM images of Ag NWs. (b) Statistic length distribution of Ag NWs extracted from Figure S1a. (c) Statistic diameter distribution of Ag NWs extracted from Figure 1b.

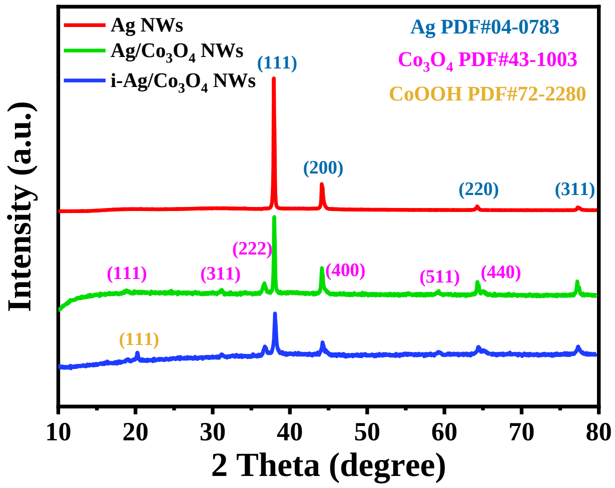

Figure S2. XRD patterns of the as-synthesized catalysts.

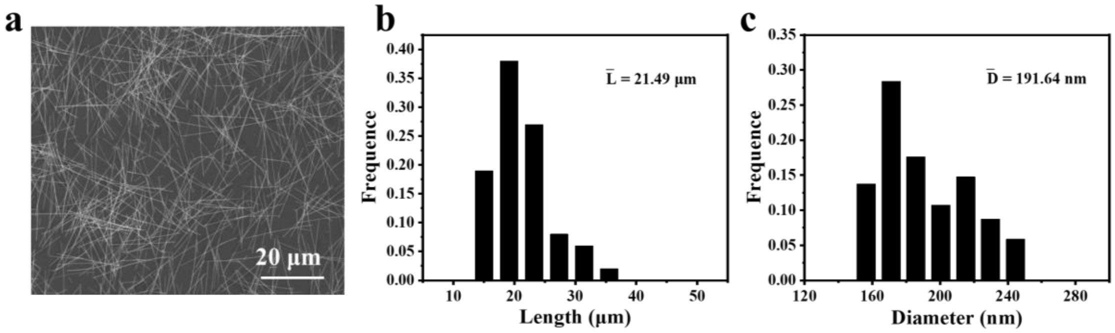

Figure S3. (a) SEM images of Ag/Co_3O_4 NWs. (b) Statistic length distribution of Ag/Co_3O_4 NWs extracted from Figure S2a. (c) Statistic diameter distribution of Ag/Co_3O_4 NWs extracted from Figure 1e.

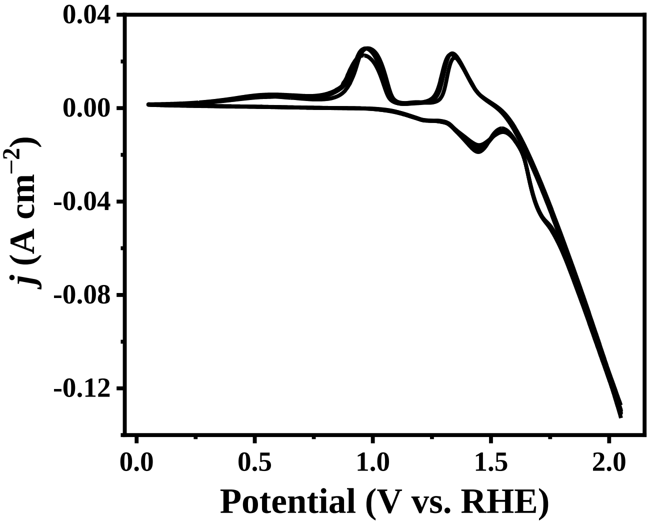

Figure S4. Cyclic voltammetry (CV) profiles of Ag/Co_3O_4 NWs in Ar−saturated 1 M KOH.

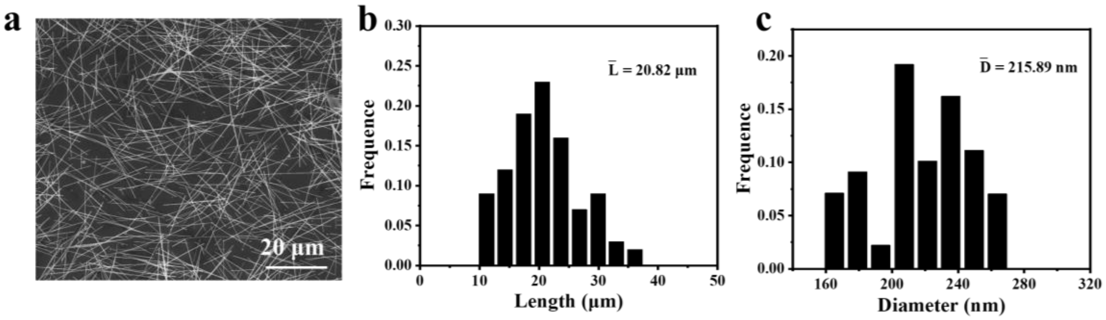

Figure S5. (a) SEM images of i-Ag/Co_3O_4 NWs. (b) Statistic length distribution of i-Ag/Co_3O_4 NWs extracted from Figure S4a. (c) Statistic diameter distribution of i-Ag/Co_3O_4 NWs extracted from Figure 1h.

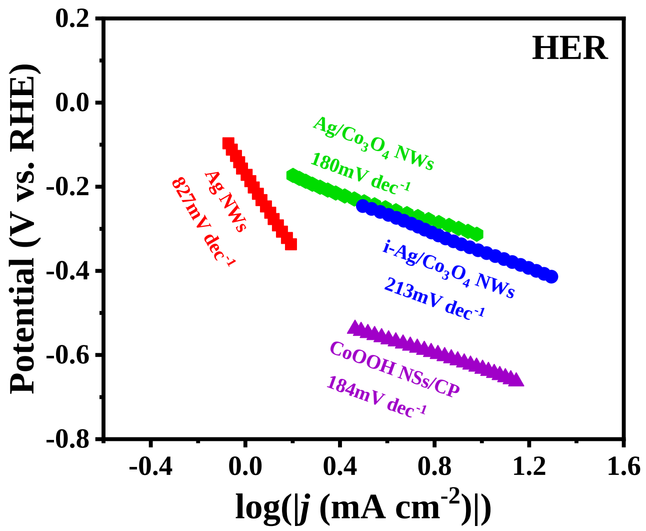

Figure S6. Tafel slopes of the catalysts for HER in 1 M KOH. The LSV curves were recorded at a scan rate of 10 mV s^{-1}.

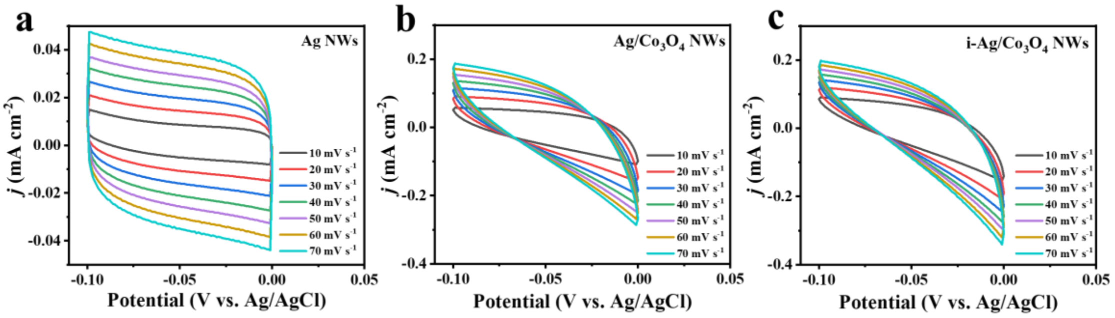

Figure S7. Cyclic voltammetry (CV) profiles were obtained on the Ag NWs (a), Ag/Co_3O_4 NWs (b), and i-Ag/Co_3O_4 NWs (c) catalysts.

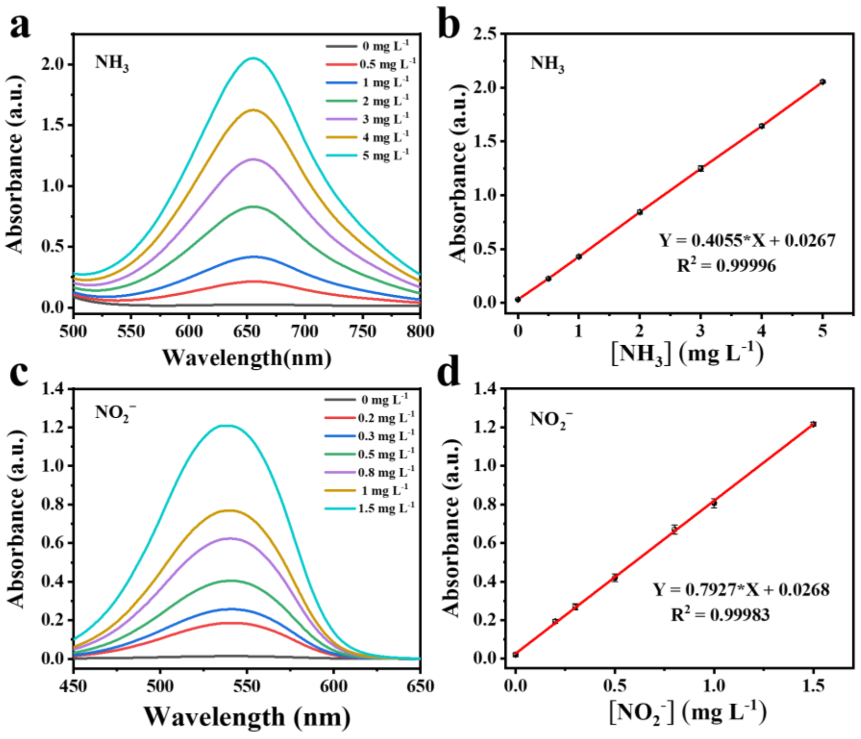

Figure S8. NH_3 and NO_2^- quantification using UV−vis absorption spectroscopy. (a-b) UV−vis absorption spectra and corresponding calibration curve for the NH_3 assay using the indophenol blue method. (c-d) UV−vis adsorption spectra and corresponding calibration curve for the NO_2^- assay.

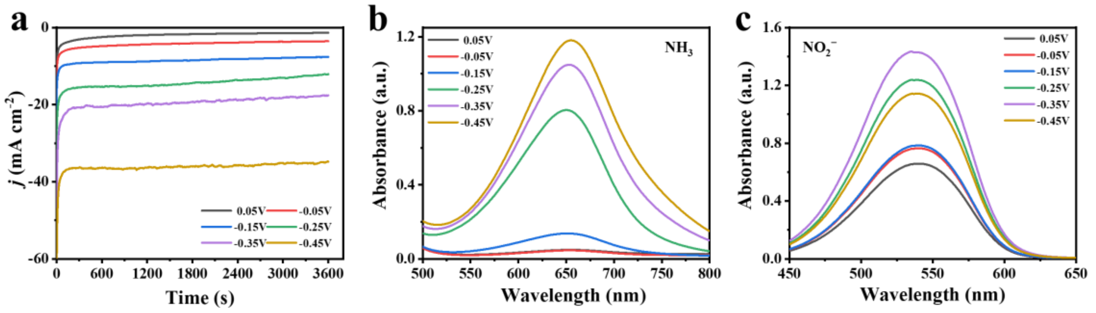

Figure S9. NH₃ synthesis performance of Ag NWs at a series of potentials. (a) Chronoamperometry curves at different potentials for 1 h in 0.1 M NO_3^- and 1 M KOH. (b-c) UV−vis absorption spectrum of NH_3 (b) and NO_2^- (c) measurements.

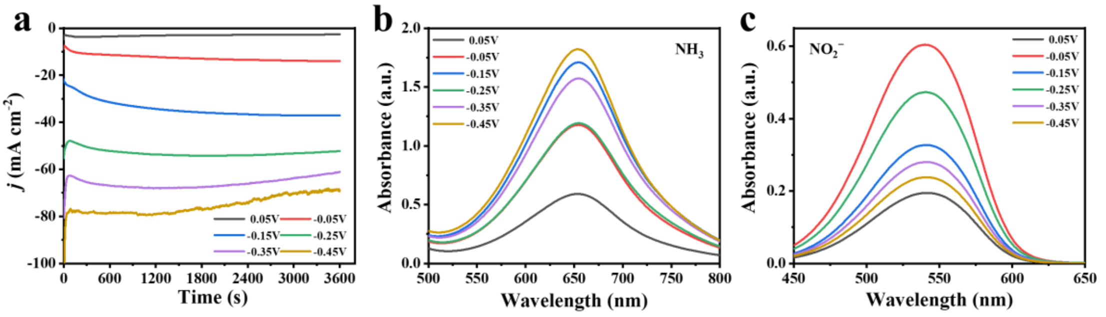

Figure S10. NH_3 synthesis performance of Ag/Co_3O_4 NWs at a series of potentials. (a) Chronoamperometry curves at different potentials for 1 h in 0.1 M NO_3^- and 1 M KOH. (b-c) UV−vis absorption spectrum of NH_3 (b) and NO_2^- (c) measurements.

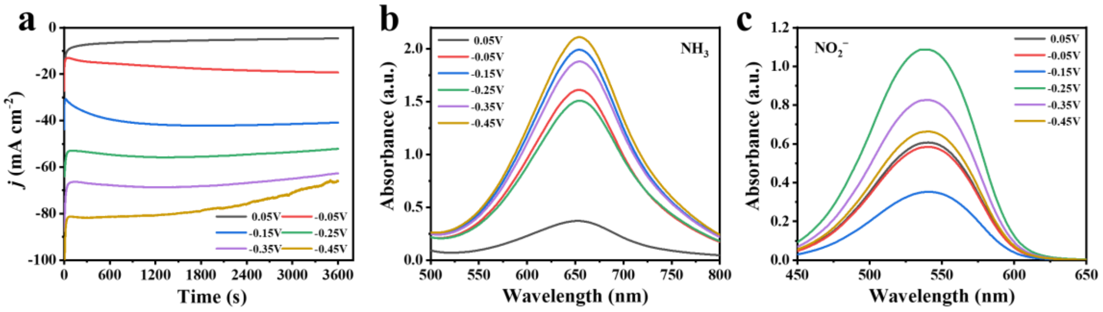

Figure S11. NH_3 synthesis performance of i-Ag/Co_3O_4 NWs at a series of potentials. (a) Chronoamperometry curves at different potentials for 1 h in 0.1 M NO_3^- and 1 M KOH. (b-c) UV−vis absorption spectrum of NH_3 (b) and NO_2^- (c) measurements.

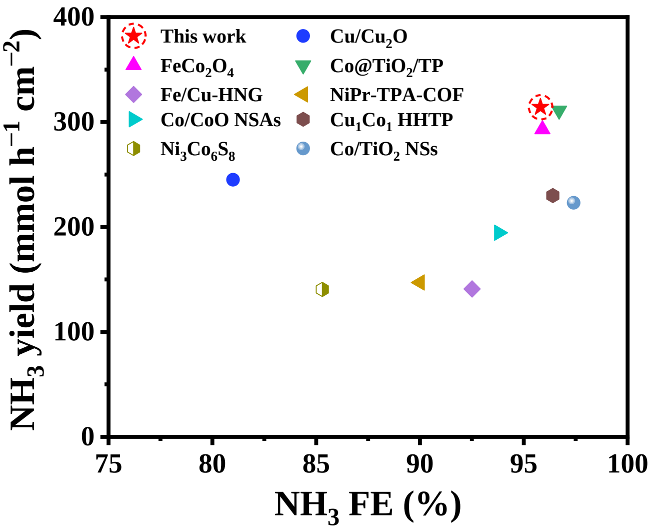

Figure 12. Comparison of NH_3 Faradaic efficiency and NH_3 yield rate of i-Ag/Co_3O_4 NWs with other reported catalysts.

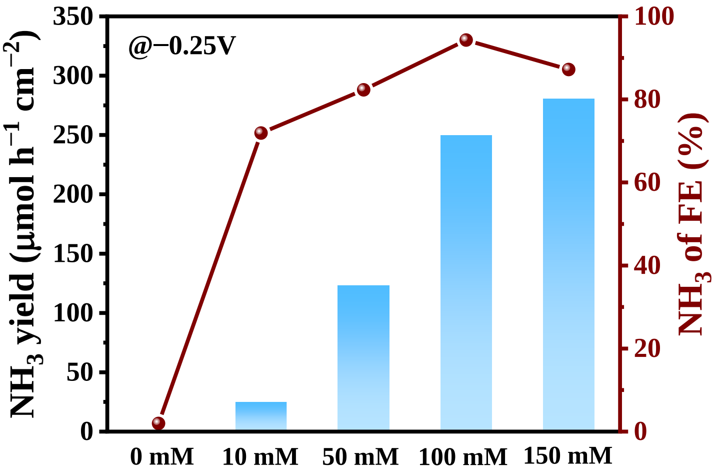

Figure S13. Comparison of the NH_3 yield rate and FE on the i-Ag/Co_3O_4 NWs catalysts at different NO_3^- concentrations at −0.25 V *vs.* RHE.

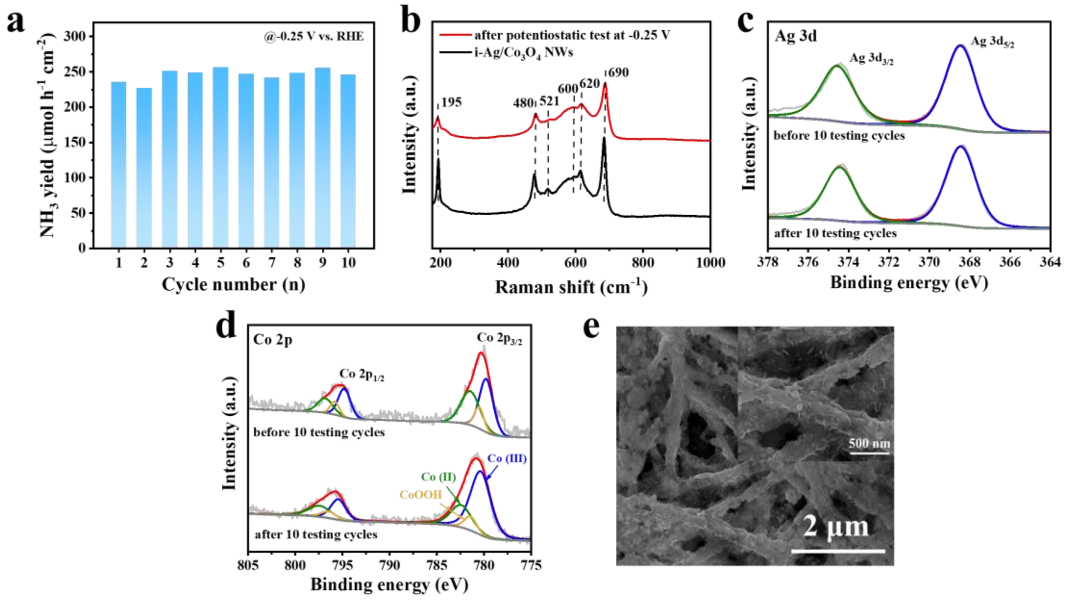

Figure S14. (a) The cycling tests of the i-Ag/Co_3O_4 NWs for the reduction tests at −0.25 V *vs.* RHE. (b) The Raman spectra. XPS spectra of (c) Ag 3d and (d) Co 2p of the i-Ag/Co_3O_4 NWs catalysts. (e) SEM images of i-Ag/Co_3O_4 NWs after 10 testing cycles.

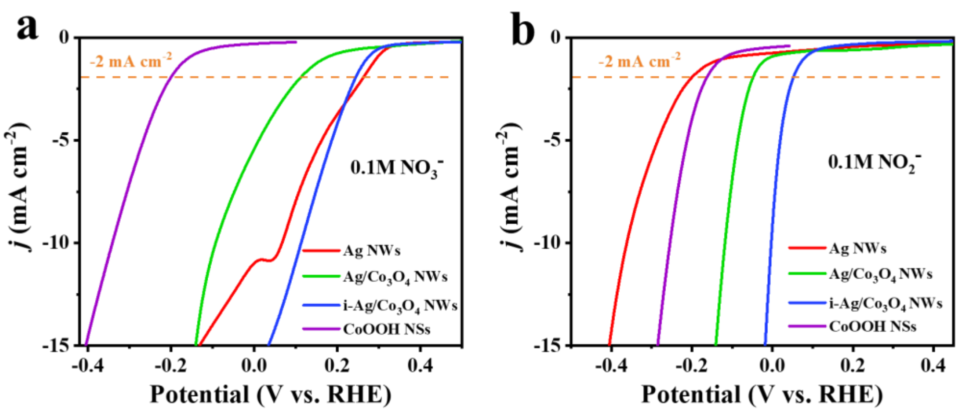

Figure S15. The LSV of the as-synthesized catalysts in the 1 M KOH solutions containing (a) 0.1 M NO_3^- and (b) 0.1 M NO_2^-. The potentials at −2 mA cm^{-2} were marked and used to compare the activities of the catalysts.

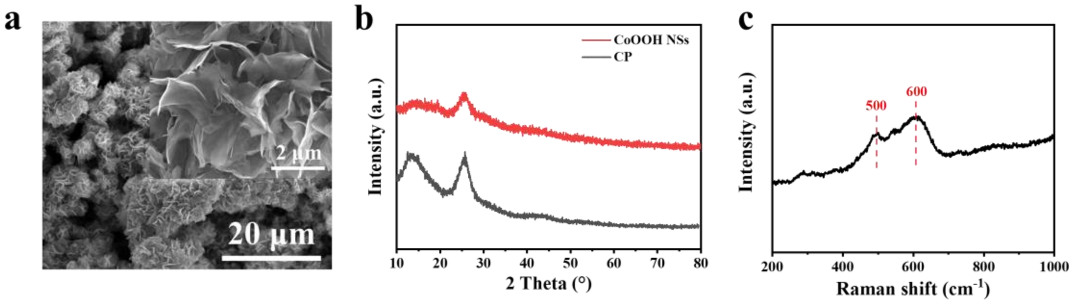

Figure S16. (a) SEM images, (b) XRD patterns, (c) Raman spectra of CoOOH NSs.

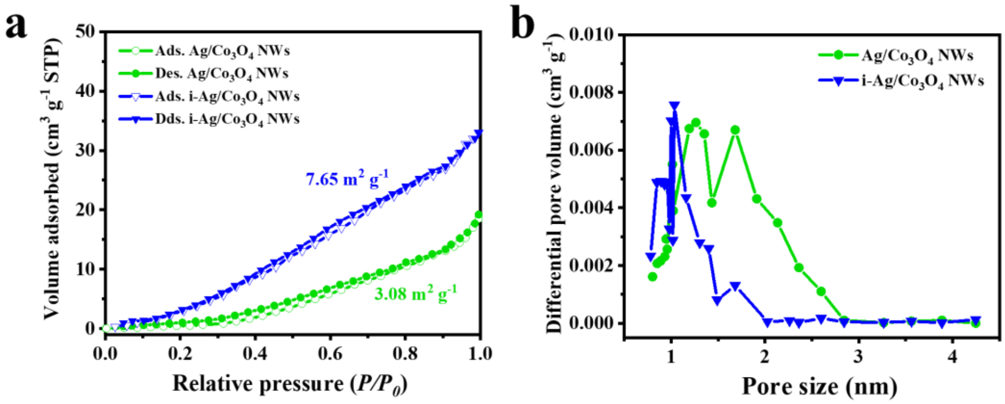

Figure S17. (a) N_2 adsorption-desorption isotherms of Ag/Co_3O_4 NWs and i-Ag/Co_3O_4 NWs. (b) Pore size distribution curves from N_2 adsorption measurements for the Ag/Co_3O_4 NWs and i-Ag/Co_3O_4 NWs.

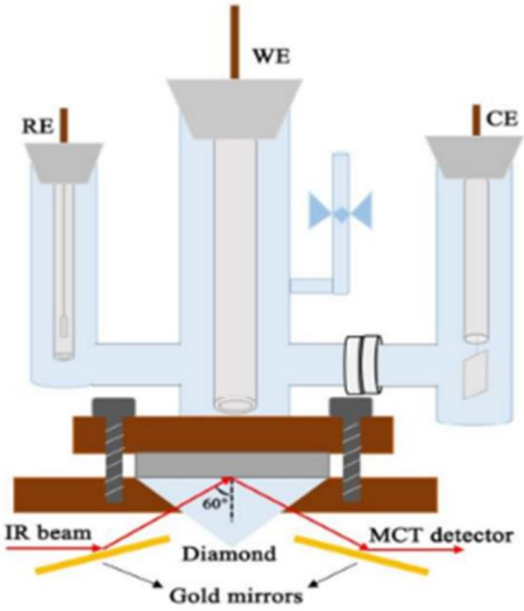

Figure S18. Schematic diagram of the in situ FTIR spectra device.

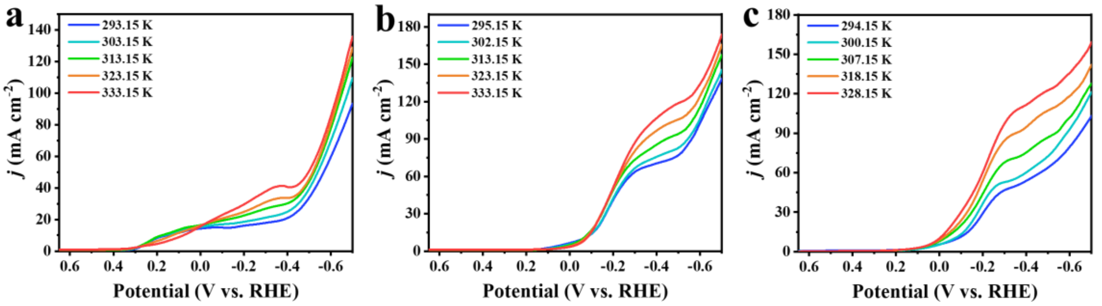

Figure S19. LSV curves of (a) Ag NWs, (b) Ag/Co_3O_4 NWs, and (c) i-Ag/Co_3O_4 NWs catalysts recorded in 1 M KOH and 0.1 M KNO_3 solution at different temperatures. These LSV results were used to calculate the apparent activation energy (E_{app}) for catalysts, as shown in Figures 3e, 3g and 3i.

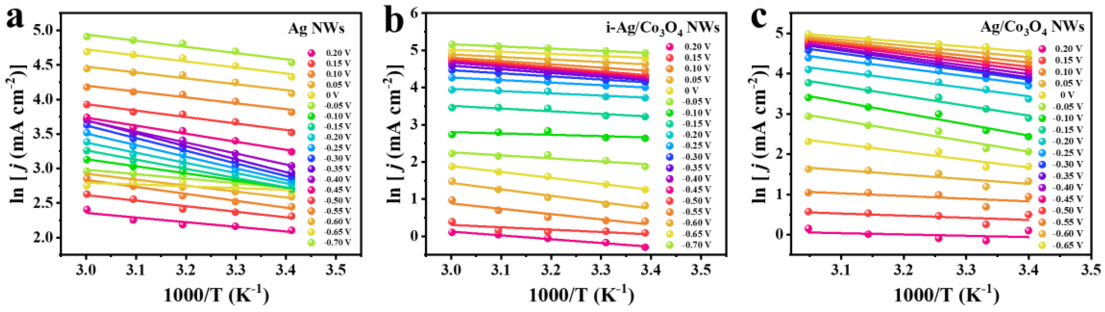

Figure S20. The logarithm of the catalytic current density plotted against 1000 times the reciprocal of the temperature (in Kelvin) to extract the apparent activation energy (E_{app}) of the NO_3RR on (a) Ag NWs, (b) Ag/Co_3O_4 NWs and (c) i-Ag/Co_3O_4 NWs catalysts at fixed overpotentials using the Arrhenius plots. The extracted E_{app} values are shown in Figures 3e, 3g, and 3i in the main text.

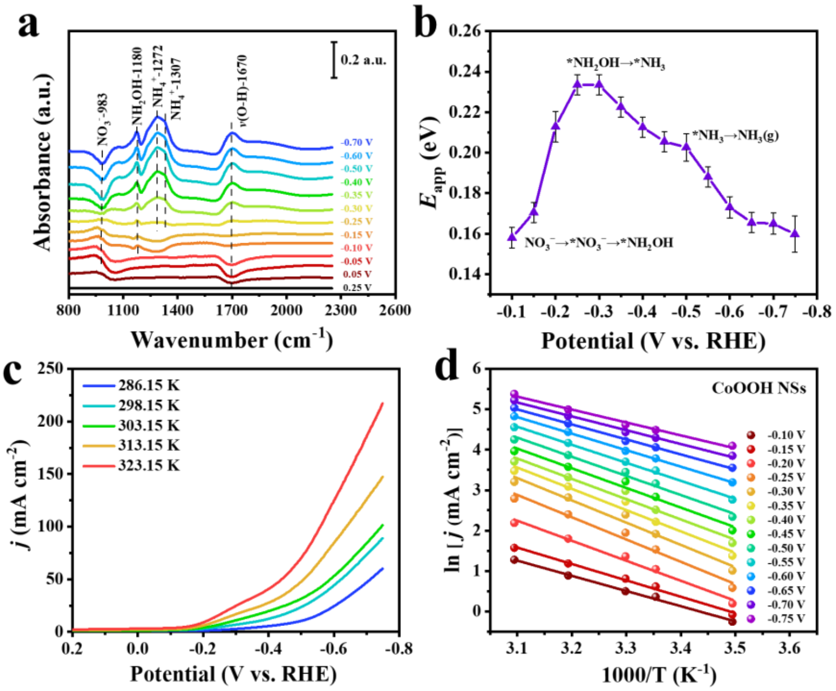

Figure S21. (a) Electrochemical in situ FTIR spectra of CoOOH NSs at different potentials in 1 M KOH and 0.1 M NO_3^- solutions. (b) CoOOH NSs catalyzed the activation energy for the NO_3RR at various potentials. (c) LSV curves of CoOOH NSs catalysts at different temperatures. (d) E_{app} of the NO_3RR on CoOOH NSs catalysts at fixed overpotentials.

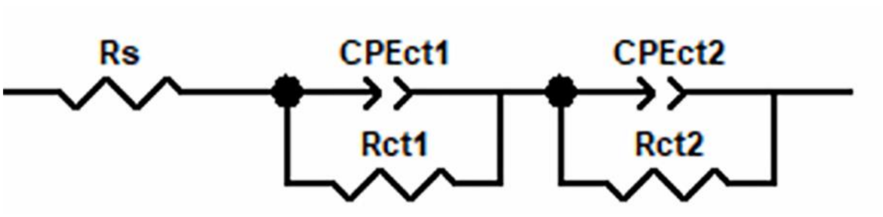

Figure S22. The equivalent circuit for modeling the measured electrochemical response. R_{ct} represents charge transfer resistance, R_s represents solution resistance and CPE_{ct} is related to double layered capacitance.

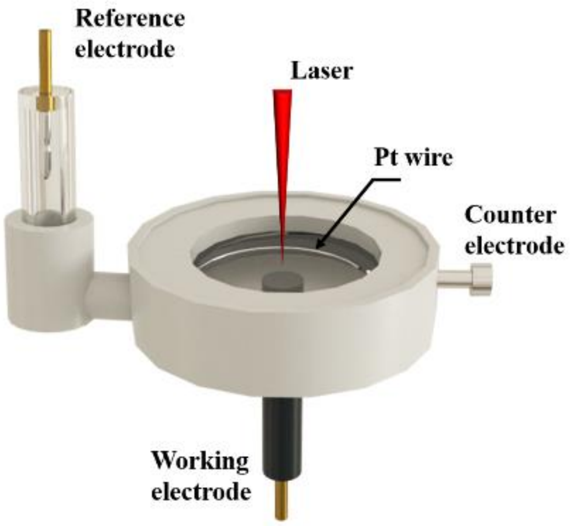

Figure S23. Schematic diagram of the homemade in situ Raman device.

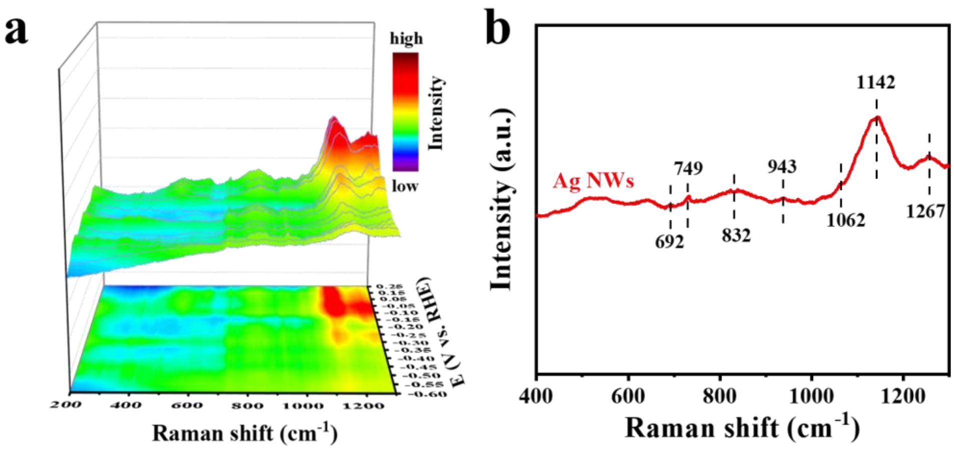

Figure S24. (a) In situ Raman spectra of Ag NWs at different applied potentials in electrolytes containing 0.1 M NO_3^- and 1 M KOH. (b) The Raman spectra of Ag NWs.

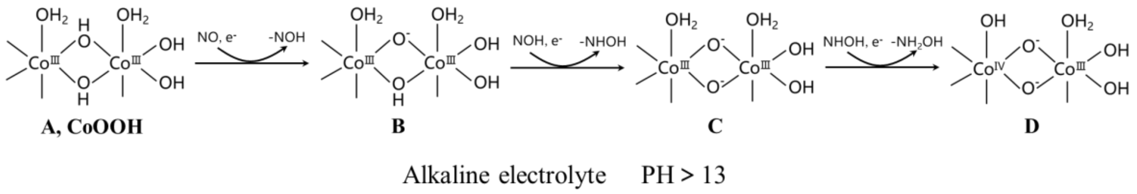

Figure S25. P roposed mechanism for the NO hydrogenation step catalyzed by CoOOH.

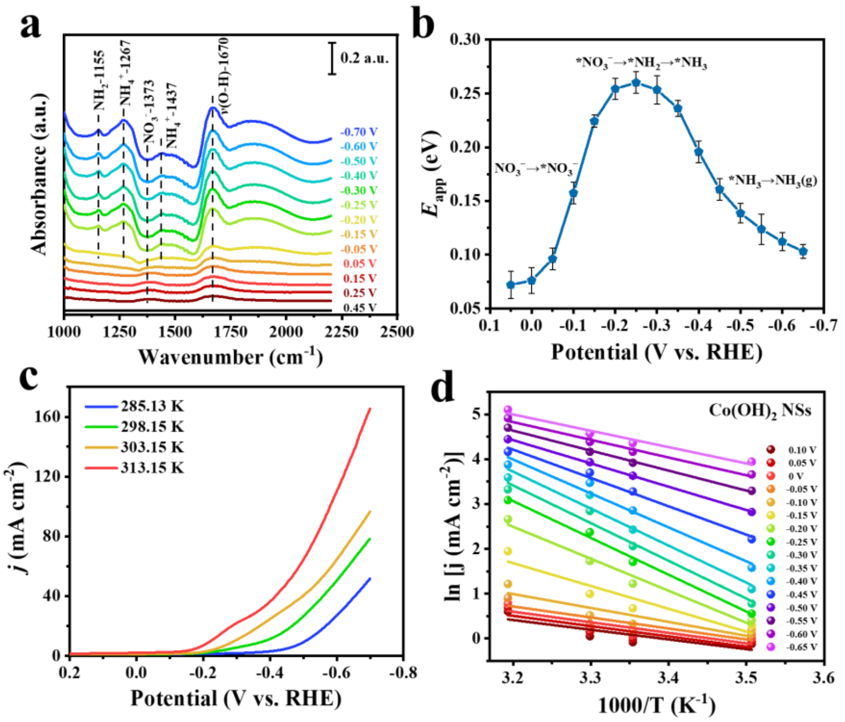

Figure S26. (a) Electrochemical in situ FTIR spectra of $Co(OH)_2$ NSs at different potentials in 1 M KOH and 0.1 M NO_3^- solutions. (b) $Co(OH)_2$ NSs catalyzed the activation energy for the NO_3RR at various potentials. (c) LSV curves of $Co(OH)_2$ NSs catalysts at different temperatures. (d) E_{app} of the NO_3RR on $Co(OH)_2$ NSs catalysts at fixed overpotentials.

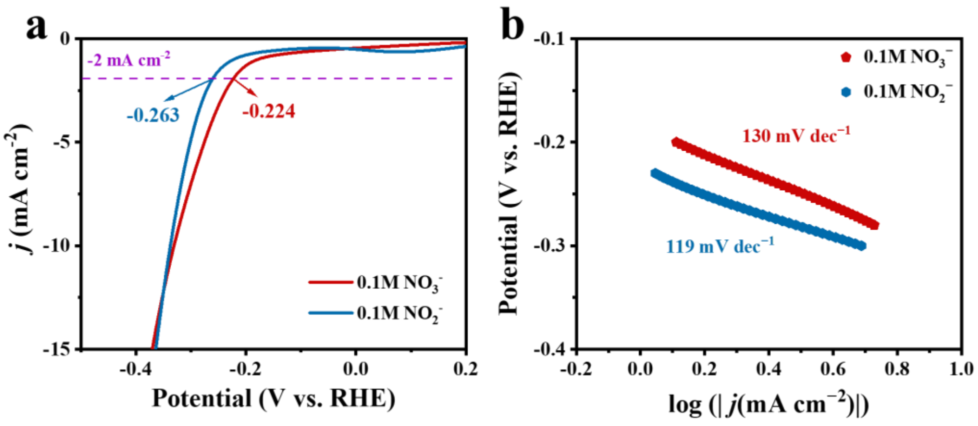

Figure S27. (a) The LSV-derived potentials at a current density of −2 mA cm^{-2} for NO_3^- and NO_2^- reduction on $Co(OH)_2$ NSs/CP catalysts. (b) The LSV-derived Tafel slopes of $Co(OH)_2$ NSs/CP.

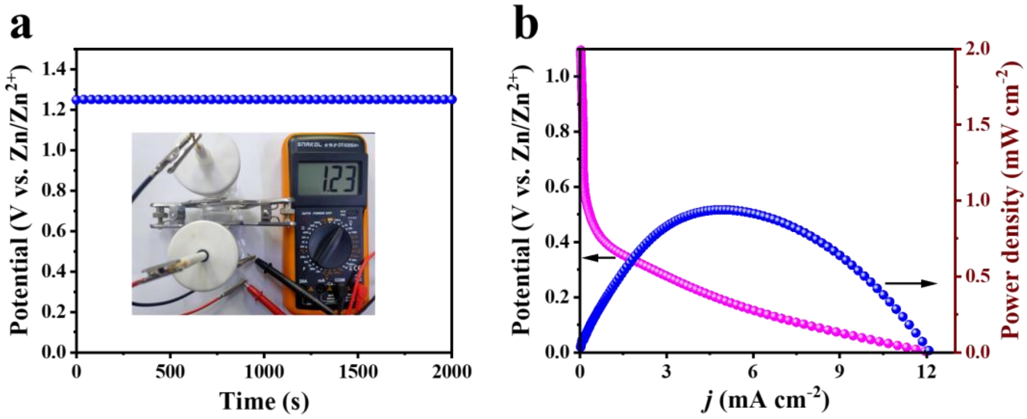

Figure S28. (a) Open circuit voltage of Ag/Co_3O_4 NWs based Zn–NO_3^- battery. (b) The discharging polarization curve for Ag/Co_3O_4 NWs based Zn–NO_3^- battery.

Supporting Tables

Table S1. Comparison of NO_3RR activity for i-Ag/Co_3O_4 NWs with other reported electrocatalysts.

Catalysts	Electrolyte	Potential (V vs. RHE)	NH_3 yield rate (μmol h^{-1} cm^{-2})	NH_3 FE (%)	Reference

**i-Ag/Co_3O_4 NWs**	1 M KOH +<br>0.1 M NO_3^-	-0.35	95.8	314.2	This work
**Cu/Cu_2O**	0.5 M Na_2SO_4 +<br>200 ppm NO_3^--N	-0.85	81	245	[5]
**$FeCo_2O_4$**	0.1M KOH +<br>0.02M KNO_3^-	-0.5	95.9	293	[12]
**Co@TiO_2/TP**	0.1 M PBS +<br>0.1 M NO_3^-	-0.7	96.7	371	[13]
**Fe/Cu-HNG**	1 M KOH +<br>0.1 M NO_3^-	-0.3	92.51	141	[14]
**NiPr-TPA-COF**	0.5 M K_2SO_4 +<br>0.1 M NO_3^-	-0.74	90	147.1	[15]
**Co/CoO NSAs**	0.1 M Na_2SO_4 +<br>200 ppm NO_3^--N	-0.64	93.8	194.5	[16]
**Cu_1Co_1 HHTP**	0.5 M Na_2SO_4 +<br>0.1 M NO_3^-	-0.6	96.4	299.9	[17]
**$Ni_3Co_6S_8$**	1 M KOH +<br>50 mg L^{-1} NO_3^--N	-0.4	85.3	140.5	[18]
**Co/TiO_2 NSs**	1 M PBS +<br>0.4 M NO_3^-	-0.72	97.4	223	[19]

Table S2. EIS parameters of Ag/Co_3O_4 NWs and i-Ag/Co_3O_4 NWs.

	Potential (V vs.RHE)	R_s (Ω)	R_{ct1} (Ω)	CPE_{ct1} (mF)	R_{ct2} (Ω)	CPE_{ct2} (mF)	R_{total} (Ω)
	0.25	2.82	430.50	0.95	1311	0.89	1741.50
	0.15	2.82	505.70	0.91	35.24	1.15	540.94
	0.05	2.81	69.9	0.87	81.30	1.01	151.20

Ag/Co_3O_4 NWs	-0.05	2.81	28.05	0.88	25.19	1.05	53.24
	-0.15	2.86	4.28	0.86	3.99	0.75	8.27
	-0.25	2.76	3.04	1.05	2.99	0.81	6.03
	-0.35	2.72	1.25	0.84	1.84	0.74	3.09
	0.25	2.52	26.22	1.09	206.10	0.87	232.32
	0.15	2.45	7.26	0.51	53.82	0.93	61.08
	0.05	2.45	0.22	0.91	25.14	0.93	25.36
i-Ag/Co_3O_4 NWs	-0.05	2.53	0.06	1.22	2.86	0.98	2.92
	-0.15	2.88	1.01	1.06	0.52	1.28	1.53
	-0.25	2.72	0.82	0.97	0.48	1.19	1.30
	-0.35	2.31	0.84	0.51	0.25	0.97	1.09

References

[1] A. Moysiadou, S. Lee, C.S. Hsu, H.M. Chen, X. Hu, J. Am. Chem. Soc. 2020, 142, 11901-11914.

[2] S.H. Ye, Z.X. Shi, J.X. Feng, Y.X. Tong, G.R. Li, *Angew. Chem. Int. Ed.* **2018**, 57, 2672-2676.

[3] Y. Zhao, R. Shi, X. Bian, C. Zhou, Y. Zhao, S. Zhang, F. Wu, G.I.N. Waterhouse, L.Z. Wu, C.H. Tung, T. Zhang, *Adv. Sci.* **2019**, 6, 1802109.

[4] J. Wang, L. Yu, L. Hu, G. Chen, H. Xin, X. Feng, *Nat. Commun.* **2018**, 9, 1795.

[5] Y. Wang, W. Zhou, R. Jia, Y. Yu, B. Zhang, *Angew. Chem. Int. Ed.* **2020**, 59, 5350-5354.

[6] J.Y. Fang, Q.Z. Zheng, Y.Y. Lou, K.M. Zhao, S.N. Hu, G. Li, O. Akdim, X.Y. Huang, S.G. Sun, *Nat. Commun.* **2022**, 13, 7899.

[7] L. Sun, B. Liu, *Adv. Mater.* **2023**, 35, e2207305.

[8] J. Huang, H. Sheng, R.D. Ross, J. Han, X. Wang, B. Song, S. Jin, *Nat. Commun.* **2021**, 12, 3036.

[9] M. Suermann, T.J. Schmidt, F.N. Büchi, *Electrochim. Acta.* **2018**, 281, 466-471.

[10] W. He, J. Zhang, S. Dieckhofer, S. Varhade, A.C. Brix, A. Lielpetere, S. Seisel, J.R.C. Junqueira, W. Schuhmann, *Nat. Commun.* **2022**, 13, 1129.

[11] R.Y. Hodgetts, A.S. Kiryutin, P. Nichols, H.-L. Du, J.M. Bakker, D.R. Macfarlane, A.N. Simonov, *ACS Energy Lett.* **2020**, 5, 736-741.

[12] J. Li, D. Zhao, L. Zhang, L. Yue, Y. Luo, Q. Liu, N. Li, A.A. Alshehri, M.S. Hamdy,

Q. Li, X. Sun, *Chem. Commun.* **2022**, 58, 4480-4483.

[13] X. Fan, D. Zhao, Z. Deng, L. Zhang, J. Li, Z. Li, S. Sun, Y. Luo, D. Zheng, Y. Wang, B. Ying, J. Zhang, A.A. Alshehri, Y. Lin, C. Tang, X. Sun, Y. Zheng, *Small.* **2023**, 19, 2208036.

[14] S. Zhang, J. Wu, M. Zheng, X. Jin, Z. Shen, Z. Li, Y. Wang, Q. Wang, X. Wang, H. Wei, J. Zhang, P. Wang, S. Zhang, L. Yu, L. Dong, Q. Zhu, H. Zhang, J. Lu, *Nat. Commun.* **2023**, 14, 3634.

[15] F. Lv, M. Sun, Y. Hu, J. Xu, W. Huang, N. Han, B. Huang, Y. Li, *Energy Environ. Sci.* **2023**, 16, 201-209.

[16] Y. Yu, C. Wang, Y. Yu, Y. Wang, B. Zhang, *Sci. China Chem.* **2020**, 63, 1469-1476.

[17] P. Liu, J. Yan, H. Huang, W. Song, *Chem. En. J.* **2023**, 466, 143134.

[18] W. Tao, P. Wang, H. Li, R. Huang, G. Zhou, *Appl.Catal. B: Environ.* **2023**, 324, 122193.

[19] Y. Xu, Y. Han, D.K. Sam, Y. Cao, *J. Mater. Chem. A.* **2022**, 10, 22390-22398.
